# Photocatalytic Degradation of Pharmaceuticals Carbamazepine, Diclofenac, and Sulfamethoxazole by Semiconductor and Carbon Materials: A Review

**DOI:** 10.3390/molecules24203702

**Published:** 2019-10-15

**Authors:** Ana S. Mestre, Ana P. Carvalho

**Affiliations:** Centro de Química e Bioquímica and Centro de Química Estrutural, Faculdade de Ciências, Universidade de Lisboa, 1749-016 Lisboa, Portugal

**Keywords:** carbamazepine, diclofenac, sulfamethoxazole, semiconductor composites, C-doping, activated carbon, CNT, graphene, CQD, biochar, sunlight-driven photocatalysis

## Abstract

The presence of pharmaceutical compounds in the environment is a reality that calls for more efficient water treatment technologies. Photocatalysis is a powerful technology available but the high energy costs associated with the use of UV irradiation hinder its large scale implementation. More sustainable and cheaper photocatalytic processes can be achieved by improving the sunlight harvesting and the synthesis of semiconductor/carbon composites has proved to be a promising strategy. Carbamazepine, diclofenac, and sulfamethoxazole were selected as target pharmaceuticals due to their recalcitrant behavior during conventional wastewater treatment and persistence in the environment, as properly reviewed. The literature data on the photocatalytic removal of carbamazepine, diclofenac, and sulfamethoxazole by semiconductor/carbon materials was critically revised to highlight the role of the carbon in the enhanced semiconductor performance under solar irradiation. Generally it was demonstrated that carbon materials induce red-shift absorption and they contribute to more effective charge separation, thus improving the composite photoactivity. Carbon was added as a dopant (C-doping) or as support or doping materials (i.e*.,* nanoporous carbons, carbon nanotubes (CNTs), graphene, and derived materials, carbon quantum dots (CQDs), and biochars) and in the large majority of the cases, TiO_2_ was the semiconductor tested. The specific role of carbon materials is dependent on their properties but even the more amorphous forms, like nanoporous carbons or biochars, allow to prepare composites with improved properties compared to the bare semiconductor. The self-photocatalytic activity of the carbon materials was also reported and should be further explored. The removal and mineralization rates, as well as degradation pathways and toxicity of the treated solutions were also critically analyzed.

## 1. Introduction

World’s population increase along with the expected economic development, are major challenges water and energy consumption. However, nowadays 800 million people do not have access to drinking water and 1000 million live without electricity [1]. Thus, all strategies that enable to maximize water reuse, and contribute to the preservation of water resources, as well as those promoting more efficient use of renewable energies (e.g., solar energy), are essential to help in minimizing water scarcity and energy demand.

Water treatment technologies upgrade is the key to face current water quality challenges. The upmost challenge for drinking water and wastewater treatment, as well as water reuse, is linked to the presence of natural organic matter (NOM) and micropollutants in raw water, the latter often related to pharmaceutical compounds (PhCs). Several PhCs partially or totally resist to conventional treatments. So, it is crucial to upgrade the design and operation conditions of wastewater treatment plants (WWTPs), and water treatment plants (WTPs) with innovative, cost- and resource-effective solutions to achieve high-quality water standards.

Some of PhCs resist conventional treatments and are also highly resistant to photolysis and/or adsorption onto solid matter leading to their high persistence in the aquatic environment. Consequently, tertiary treatments are fundamental to assure the removal of these recalcitrant PhCs. These end-of-line treatments cover a large range of technologies: UV disinfection, ozone or chloride, and advanced water treatment technologies. This last class gathers activated carbons adsorption, membranes filtration, advanced oxidation processes (AOPs), and also combinations of two or more technologies. The use of technologies based on adsorption or membranes transfers the pollutant from the water to the pore structure of the adsorbent material or to the reject of the membrane filtration, originating a residue that still requests further treatment or disposal in a landfill. On the contrary, ideal AOPs allow total mineralization of the pollutant to CO_2_, water and inorganic compounds, or at least degrade the pollutant into more innocuous compounds.

AOPs can be broadly defined as aqueous phase oxidation methods based on the formation of highly reactive species, such as (mainly but not exclusively) hydroxyl radicals (OH^●^), leading the target pollutants decomposition [2]. Extensive research has been carried out on the use of AOPs to treat industrial and urban effluents. AOPs are divided into homogeneous and heterogeneous processes when considering their occurrence in a single phase or not, respectively. Depending on the methodology of OH^●^ generation, AOPs can also be classified as chemical, electro-chemical, sono-chemical, and photo-chemical processes [3].

PhCs are a very large group of organic micropollutants of emergent concern. Thus remediation studies usually focus on a small group of PhCs classified as recalcitrant to conventional water treatment technologies and representative of distinct therapeutical classes and chemical functionalities. Following this rational three PhCs were selected to be addressed in this review: carbamazepine (CBZ) an antiepileptic, diclofenac (DCF) an acidic anti-inflammatory and analgesic, and sulfamethoxazole (SMX) an antibiotic (Table 1).

CBZ is an antiepileptic drug used to control seizures, is also used to relief neuralgia, and as an antidepressant in bipolar depression. DCF is a popular non-steroidal anti-inflammatory drug, having also antiarthritic and antirheumatic properties. In 2008, Zhang et al. estimated 1014 ton CBZ and 940 ton DCF consumption per year in accordance with IMS health data reporting 942 tons of CBZ and 877 tons of DCF sold in 2007 [11]. In 2013, the European Commission established the first watch list with 10 substances that may pose a significant risk to, or via, the aquatic environment (Directive 2013/39/EU). DCF was included in this list and maintained in next revision (Directive 2015/495/EU). The most recent revision removed DCF considering that sufficient high-quality monitoring data was already available (Directive 2018/840/EU). SMX is an extensively used antibiotic from the sulfonamide class of antibacterial compounds. It is used in clinic for the treatment of respiratory tract infections, severe urinary tract infections, and enteric infections [12], is also used in veterinary (to treat infections or for preventive care in aquaculture) and as herbicide [13].

In this context, this work starts reviewing the literature supporting the relevance of CBZ, DCF, and SMX as representatives of PhCs with recalcitrant behavior in water treatment and in natural photodegradation processes. Secondly, the use of semiconductor and carbon materials for the photocatalytic degradation of these PhCs will be addressed, highlighting the role of carbon materials, in particular for the improved sunlight harvesting.

## 2. Pharmaceutical Compounds as Pollutants of Emergent Concern

PhCs are essential in assuring high human health standards and well-being and are also largely employed in veterinary, being ubiquitous chemicals in modern societies. However, it is proven that the high consumption patterns of PhCs associated with their high chemical stability and low biodegradability, contributed to their widespread release in the aquatic environment (mainly for those highly soluble). Given the biological activity and recalcitrant behavior of several PhCs, they constitute a major treat firstly to aquatic life and consequently to the whole food chain. Besides the public health concerns regarding short and long-term toxicological effects related to the exposure to trace amounts of PhCs trough potable water and food chain, the environmental perspective of this problem is also a relevant topic.

The first report on PhCs occurrence in the environment dates from 1965 when Stumm-Zollinger and Fair addressed the presence of steroid hormones in the effluents of the U.S.A. WWTPs to understand their biodegradation in WWTP, and in receiving water bodies that could eventually supply drinking water to households [14]. In the 1970s clofibric acid, salicylic acid, and chlorophenoxyisobutyrate (PhCs and metabolites) were detected in the U.S.A. wastewater effluents [15,16]. Nowadays, PhCs are detected in all sorts of environmental waters worldwide, and some of them are part of a watch list for EU-wide monitoring to access their risk for aquatic environment and support future prioritization (2018/840/EU).

### 2.1. CBZ, DCF, and SMX Detections in Wastewater and Environmental Water

Since the early monitoring studies in 1980s and 1990s CBZ, DCF, and SMX have been detected in wastewater samples and in environmental waters in the ng/L–μg/L range (Table 2). Between 1996 and 2000 Heberer [17] monitored 24 PhCs in sewage treatment plants (STPs) and environmental waters from the urban area of Berlin and CBZ, DCF, and SMX were the compounds detected at the higher concentrations. The results allowed to conclude that only 8% CBZ and 17% DCF were removed during wastewater treatment. Consequently, not surprisingly, concentrations up to 1.1 μg/L CBZ and 0.6 μg/L DCF were detected in surface water of the Berlin urban area [17]. In 2001, Ternes reported negligible CBZ removal in a municipal STP, the treatment of DCF was more effective (69%) [18]. CBZ and DCF were also detected in the same concentration range in STPs effluents and environmental waters from other European countries and Brazil [19,20,21,22,23,24]. Frist monitoring studies also report the detection of SMX in STPs effluents at ≈0.9 μg/L but reaching a maximum of 2.0 μg/L [21,25].

In 2010, Loos and co-workers analyzed 35 compounds (pharmaceuticals, pesticides, perfluorinated acids, benzotriazoles, hormones, and endocrine disrupters) in more than 100 river water samples from 27 European countries. Among all PhCs CBZ was the most frequently detected compound (95%) and at the highest concentration level (0.308 μg/dm^3^ for percentile 90%, and a maximum of 11.561 μg/dm^3^) [29]. Also DCF and SMX were detected with a frequency above the average frequency for all the compounds under study (61%), i.e., DCF was detected in 83% of the samples at concentrations of 0.043 μg/dm^3^ in 90% percentile, and maximum values of 0.247 μg/dm^3^, while SMX was detected in 75% of the analysis at concentrations of 0.104 μg/dm^3^ in 90% percentile, and maximum values of 4.072 μg/dm^3^ [29]. A survey on the occurrence of 59 polar organic persistent pollutants in 164 locations of European ground water (23 countries) identified 21 relevant compounds, among which CBZ (detection frequency 42%, max. 0.39 μg/dm^3^) and SMX (detection frequency 24%, max. 0.038 μg/dm^3^) [30]. The study also showed that CBZ was frequently detected above the European ground water quality standard for pesticides (0.1 μg/dm^3^) [30].

The results of a systematic study regarding the fate of CBZ in eleven Austrian STPs conducted by Clara et al. in 2004, revealed that this antiepileptic medicine consistently resisted to degradation and adsorption during conventional wastewater treatment, and also during soil passage and subsurface flow, thus allowing to propose CBZ as an anthropogenic marker for detecting wastewater in the aquatic environment [31].

In 2008, Zhang et al. reviewed literature data on CBZ and DCF removal during wastewater treatment as well as their occurrence in water bodies [11]. According to monitoring data in WWTPs (1998–2007), CBZ is considered persistent since 70% of the studies reported only 0 to 10% removal of this PhC. For DCF the removal efficiencies reported varied between 0 and 80%. The justification for the distinct behavior can be related to the high resistance of CBZ to biodegradation at low concentration while DCF can be biodegraded at specific conditions (i.e*.,* anoxic biofilm reactor, acidic conditions) [11]. Phototransformation during sunlight irradiation in WWTPs cannot be disregarded as a removal route. However, in deionized water CBZ presents very high stability with half-life of approximately 100 days while DCF and SMX present values two orders of magnitude lower (5.0 and 2.4 days, respectively) [11,23]. In real wastewater matrices the presence of organic matter that can behave as photosensitizers or inner filters must also be considered. In fact, humic acids act as inner filters for CBZ and DCF lowering their degradation, but in the case of SMX behave as photosensitizers promoting its phototransformation [11,23].

The recalcitrant nature of some PhCs during conventional wastewater treatment can be justified in light of their properties. According to Joss et al. [6], the biological degradation constants (*k*_biol_, Table 1) of CBZ and DCF are indicative of less than 20% removal during wastewater treatment with conventional activated sludge (CAS) or membrane bioreactor (MBR) technologies, supporting the systematic low biodegradability during secondary treatment. Regarding CBZ, DCF, and SMX also, no sorption onto activated sludge is expected to occur due to distribution coefficient water/sludge values (*k*_d_, Table 1) much lower than reference value for significant sorption (0.500 L/g_ss_) [11]. Volatilization could be another route to the removal of these PhCs during wastewater treatment but their low Henry constant values (*k*_H_, Table 1) indicate that they are nonvolatile compounds.

A very recent study classified 13 PhCs, including DCF and SMX, as priority PhCs in the aquatic environment of China, according to the proposed ranking system that is supported on three criteria (occurrence, exposure potential, and ecological effects). [32]. DCF and erythromycin presented the highest predicted environmental risk. This prioritization is in line with other ranking studies since DCF is considered a priority PhC in eight reports, and SMX in six of the works [32]. In fact, Gou et al. identified 13 PhCs (including CBZ and DCF) and three metabolites as presenting high risk score since their estimated exposure is higher than predicted no-effect concentration (PNEC) [33]. Besse et al. calculated the risk quotient ratios with available ecotoxicity data of 76 PhCs and their metabolites and, according to the conditions required by the European Medicine Agency (EMEA) guidelines of 2006, CBZ, DCF, and SMX are three of the six PhCs identified as presenting environmental risk [34]. The Global Water Research Coalition (GWRC) consolidated data from 25 literature studies on 153 PhCs and, according with the seven more relevant criteria, CBZ, DCF, and SMX were identified as the top three PhCs of the group of 10 high priority PhCs [35]. In fact, these three PhCs present the highest number of occurrences in the literature (15, 12, 13, respectively) being the only ones that fulfil all the relevant criteria (i.e, regulation, consumption/sales, physicochemical properties, toxicity, occurrence—wastewater, river water, ground water, drinking water—, degradability/persistence and resistance to treatment) [35].

### 2.2. Photodegradation

As mentioned previously, the occurrence of CBZ, DCF, and SMX in environmental waters worldwide results from the low effectiveness of conventional water treatments as barriers for these PhCs, due to their high chemical stability and low biodegradability. Once in the environment, the fate of these PhCs in the water-cycle depends on their properties namely their hydrophobic/hydrophilic character that will determine their interactions with water, dissolved species, and solids, as well as, their stability under sunlight irradiation.

**Figure 1:**
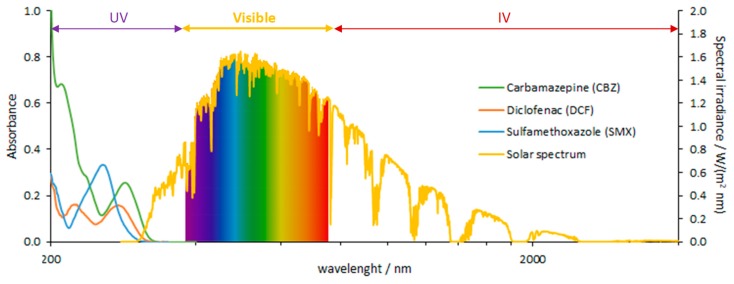
UV absorption spectra of CBZ, DCF, and SMX (5 mg/L solutions in inorganic matrix at pH 7.1) versus sunlight spectrum at sea level (ASTM G173-03 Global, [36]).

Photodegradation at the top level of surface waters may be a natural elimination pathway for recalcitrant PhCs in environmental waters. Direct and indirect photochemical processes can occur depending on the PhC nature, water matrix, and solar irradiation spectrum. Direct photolysis of an organic compound only occurs when the absorption spectrum of the compound overlaps the intensity spectrum of the incoming irradiation, resulting in the electronic excitation of the molecule that promotes its phototransformation. In indirect processes, photoactive compounds—photosensitizers—absorb sunlight producing reactive species such as hydroxyl radicals (OH^●^) that can induce PhCs degradation. According to the UV spectra (Figure 1) it is possible to conclude that only a very small fraction of sunlight (i.e., UV-A and UV-B) is expected to promote direct photolysis of CBZ, DCF, and SMX.

Doll and Frimmel [37] evaluated the fate of CBZ in surface waters under natural and simulated solar light, concluding that although CBZ absorbs most of the incoming radiation compared with the other PhCs, it presents the lowest degradation rate constants. In real waters with low concentrations of NOM photodegradation of CBZ can be favored (indirect pathway by NOM-induced reactions), but in high concentration, NOM has a detrimental effect acting as the inner filter (radical scavenger and/or as precursor of reactive species). The presence of other PhCs also decreases the CBZ degradation rate (competitive inhibition) [37].

Andreozzi et al. studied the photodegradation of CBZ (8.0 × 10^−6^ mol/L) in bi-distilled water at pH 5.5 during solar light exposure determining a pseudo-first order kinetic constant of 5.7 × 10^−3^ h^−1^ and a half-life time of 121.6 h, while in spiked natural water CBZ half-life time increased to 907 h [38]. Assays in the presence of nitrates reveal that these anions act as photosensitizers reducing CBZ half-life time to 69 h–11.2 h (5.0 × 10^−4^–1.5 × 10^−2^ g/L). On the other hand, dissolved humic acid increased photodegradation half-life time of CBZ to 233.7 h possibly related to the reduction of direct photodegradation due to strong UV absorption of humic acid that acts as inner filters [38]. The laboratorial study developed by Yamamoto et al. also pointed out relatively high resistance of CBZ (20–100 µg/L) to solar light photodegradation with half-life times of 84 h, in August 2006, and 2100 h, in May 2007 [39].

Lam et al. studied the aquatic persistence of eight PhCs in sunlight-exposed pond water identifying CBZ and SMX as the most persistent with mean half-life times of 82 days and 19 days, respectively, due to their resistance to both biodegradation and photolysis [40].

The low photodegradability of CBZ is certainly related to its low quantum yield. In fact, according with the experimental quantum yields for the direct photolysis of PhCs in bi-distilled water at pH 5.5, CBZ has one of the lowest values (4.77 × 10^−5^ under sunlight) [23]. For SMX the value obtained under lamplight was 4.29 × 10^−3^ mol/Einstein and for DCF the results reported are 3.13 × 10^−2^ mol/Einstein under lamplight, and 3.37 × 10^−2^ mol/Einstein under sunlight [23]. Chiron et al. determined an average quantum yield of 1.5 × 10^−4^ mol/Einstein for CBZ direct photolysis in Milli-Q water under medium-pressure mercury lamp (highest contribution from 313, 366, 406 nm) [41]. In surface water at pH 7 quantum yields of CBZ irradiated with medium-pressure (205–500 nm) and low-pressure (253.7 nm) mercury lamps are slightly higher (0.6 × 10^−3^ and 2.3 × 10^−3^ mol/Einstein, respectively) [42].

The photodegradation of CBZ (2.1 × 10^−4^ mol/L) in artificial river and estuarine water (with humic acids, Fe(III), NO_3_^−^, and Cl^−^) was studied by Chiron et al. who found a decrease in photodegradation rate with increasing pH, which was attributed to the distinct iron species in solution [41]. At acidic pH values CBZ photodegradation occurs via dissolved Fe(III) that originates OH^●^ while at higher pH values Fe(III) colloids are predominant and produce lower amounts of OH^●^ but are able to induce charge-transfer reactions, this will not be pure photodegradation processes since from the Fenton process it is known that iron can catalyse the degradation. However, under neutral to basic conditions the presence of chlorine enhance CBZ photolysis most probably due to the interaction between Fe(III) colloids and Cl^−^ leading to the formation of Cl_2_^●−^ that albeit presenting slower degradation than OH^●^ would be faster than charge-transfer processes occurring on the surface of Fe(III) colloids [41]. After 8 h irradiation, the toxic, mutagenic and carcinogenic acridine (major intermediate of CBZ direct photolysis) accounted for 10% of the initial carbamazepine species [41]. Since this transformation product has greater health and environmental risk than the pristine compound more effective degradation routes are needed.

In 2008, Matamoros et al. evaluated the photodegradation of four PhCs, including CBZ (10–40 mg/L), under simulated solar light and sunlight, in river water and sea water, as well as, distilled water for getting information regarding photodegradation pathways [43]. To assure irradiation close to real conditions, the authors used quartz vessels transparent to UV light. The results pointed out CBZ as the compound most resistant to photodegradation since in Milli-Q water it presented 38.5 h of half-life time, and in two river water or sea water, the value decreased to 8.25–14.4 h, revealing the role of DOC in indirect photodegradation. In the assays performed under sunlight the half-life time in river water was eight times higher (67.4 h) compared with the value obtained under simulated conditions, being this decreased explained by the daily light variation.

Early studies by Buser et al. [22] reported a great decrease in DCF concentrations from a major tributary to a lake, to the outflow of the same lake (inflow from 0.005 to 0.370 μg/dm^3^ and outflow from <0.001 to 0.012 μg/dm^3^) estimating more than 90% DCF removal most likely due to photodegradation (not detected in sediments and laboratory experiments corroborate negligible absorption onto sediment particles). Lake water spiked with DCF presented minimal chemical and biological degradation under dark, however once exposed to sunlight a fast photodegradation occurred attaining the half-life time in less than 1 h of irradiation, most likely via direct photolysis [22]. In a further study the same authors were able to identify photodegradation products, namely 8-chlorocarbazole-1-acetic acid that was detected in all the experiments and photodegrades faster than DCF, and concludes that photochemical degradation is the predominant elimination process for DCF in the lake [44].

Literature studies report decomposition quantum yields for DCF from 0.031 to 0.22 mol/Einstein in the laboratory, in agreement with those determined in environmental conditions (0.094 and 0.13 mol/Einstein) [45]. According with simulated profiles and field measurements in a lake (three-month period) Tixier et al. estimated an average elimination rate of ≈2 h^−1^ (0.082 days^−1^) for direct DCF photolysis which is in accordance with the model simulation based on field data (overall elimination rate constant ≈2.1 h^−1^ (0.088 days^−1^), and eight days of half-life time), proposing this process as the main elimination route for DCF in environmental water [46]. The same study classified CBZ as fairly persistent with an overall elimination rate constant of ≈0.26 h^−1^ (0.011 days^−1^), and 63 days of half-life time [46].

Photodegradation profiles of DCF and SMX under artificial sunlight irradiation in dechlorinated tap water at pH 8 (mimicking surface water without bacteria or suspended solids) reveal that DCF, with a half-life time of 0.40 days, was by far much more sensitive to photolysis than SMX, which needed 54 days to decrease half of the initial concentration [47]. Although SMX has relatively low absorption in the UV-B region above 290 nm, according to the studies of Moore and Zhou in the mid-1990s, the susceptibility of this sulfonamide antibiotic to photodegradation is highly dependent on the solution pH [12,48]. In fact, under UV light the pseudo-first-order kinetic constant is higher than 0.15 min^−1^ at pH < 4, and lower than 0.025 min^−1^ at pH ≥ 7, with the inflection point matching the p*K*_a2_ value [48]. Also, the quantum yield of SMX photodegradation determined under UV irradiation is 0.47 mol/Einstein at pH 3 and only 0.084 mol/Einstein at pH 9 [48].

The complex SMX speciation profile allows concluding that while the nonionized form is extremely susceptible to photodegradation under UV or sunlight irradiation, the anion form, predominant at pH 5.6 or higher (pH values of wastewater effluent and environmental water), is more stable. The high photostatibility of the SMX anionic form offers and explanation to the slower and less effective photolysis of SMX in seawater compared with deionized water, both at natural pH (4.8 and 8.1, respectively) [49] or in deionized water with pH values increasing from 3 to 10 [50]. This behavior is a strong indication that SMX ersistence in the environment is most likely due to its resistance to photodegradation in slightly basic conditions. The presence of natural water constituents (i.e., fluvic acids and suspended solids) were reported to have a negative effect on the photodegradation of SMX (1–10 mg/L), in the case of fluvic acid at 50 mg/L the half-life is five times higher compared to deionized water [50]. The type of organic matter is also relevant for the photolysis of SMX, Ryan et al. verified that effluent organic matter (EfOM) enhanced SMX photodegradation, while NOM had no effect, probably due to the higher concentration of nitrates in EfOM, which act as photosensitizers for the production of hydroxyl radicals, and also due to the presence of triplet excited state organic matter in EfOM [51].

Although acidic pH favors SMX photodegradation, the mineralization degree is negligible due to the formation of abundant transformation products [49,52]. 4-amino-N-(5-methyl-2-oxazolyl)benzenesulfonamide is the main photodegradation product identified, and although presenting the same empirical formula of SMX suffered a photoisomerization reaction of the isoxazole ring [12,40,49]. Trovó et al. evaluated the acute toxicity of SMX photodegradation products for *Daphina magna* and *Vibrio fischeri* concluding that the first was the most sensitive organism (from 60% to 100% immobilization) [49]. Since SMX was totally removed after 30 min irradiation toxicity was attributed to the degradation products. Gmurek et al. also studied the toxicity of the long-term SMX phototransformation products under simulated solar light towards *Vibrio fischeri* growth and luminescence [52]. Regardless of the irradiation time, the bioluminescence inhibition of the mixture of transformation products retained the toxicity of the parent SMX solution. Regarding growth inhibition, the increase of irradiation times shifted the curve to higher concentrations but the growth inhibition decrease was not proportional to the degradation of the SMX entities [52].

## 3. Photocatalysis

In 1972, Fujishima and Honda discovered the photoelectrocatalytic water splitting by using TiO_2_ electrodes [53] and paved the way for more efficient use of light in chemical processes. Since then photocatalysis has rapidly expanded for a large range of environmental and energy applications [54,55,56,57,58,59,60,61], is considered a potential solution to the worldwide energy shortage and for counteracting environmental degradation [60]. As stated by Fujishima and co-workers, in a time-scale of ten years the basic scientific knowledge on TiO_2_ photocatalysis allowed the development of a technological field reaching the real large-scale industrial exploitation [62], is thus a great example of fundamental science solving real word challenges.

More efficient use of the solar spectrum for an effective photodegradation of persistant PhCs as is the case of CBZ, DCF, and SMX can be achieved by applying materials with photocatalytic properties, as is the case of semiconductors. As previously mentioned TiO_2_ is by far the most popular photocatalyst due to its unique properties: inertness, high photostability and photoactivity, cost-efficiency and also non-toxicity to humans and environment [61]. Other metal oxides (e.g., ZnO, WO_3_), and their derivatives have also been explored for the degradation of organic compounds [63]. Besides the classical metal oxide semiconductors, the pioneering work by Wang et al. in 2009 reported the H_2_ evolution by metal-free polymeric graphitic carbon nitride (g-C_3_N_4_) under visible light [64], thus proposing the first metal-free photocatalyst. Since then these organic solids—g-C_3_N_4_ and derivatives, as well as other conducting polymers like polyaniline (PANI), polyacetylene, polythiophene, polypyrrole, poly-*p*-(phenylenevinylene)—proved to be promising visible-light-responsive organic photocatalysts for several key reactions (e.g., photochemical water splitting, oxidation reactions, CO_2_ mitigation, pollutant degradation and bacterial disinfection) [65,66,67,68], as well as, playing important roles in biomass conversion, i.e., sustainable chemistry processes [66].

Photocatalysis is initiated when a semiconductor absorbs a photon (*h*υ) with energy equal to, or greater than, the band gap (E_g_) of the semiconductor, causing the excitation of an electron (e^−^) from the valence band (VB) to the conduction band (CB), which leads to the formation of an electron-hole (e^−^/h^+^) pair (reaction (1)). These photogenerated charge carriers (e^−^/h^+^) can recombine or migrate to the surface of the catalyst. Recombination can occur (i) in the bulk of the semiconductor, (ii) and/or mediated by surface species, (iii) or adsorbed molecules. When charge carriers migrate to the surface of the photoactive material and are scavenged by the species adsorbed onto the surface of the semiconductor, they induce the redox reactions of the adsorbed species. The competition between the fast kinetic of recombination and slow kinetic of charge separation and transfer (i.e., migration) to the surface of the material determines the efficiency of the photocatalytic process. Consequently, the first requisite to boost the photo-oxidation of the target pollutant is to prevent the e^−^/h^+^ recombination. The photo-generated hole is a strong oxidizing agent that can directly react with the target pollutant or with water to produce hydroxyl radicals (OH^●^) (reaction (2)), the former occurs when the redox potential of the pollutant is less negative than that of semiconductor valence band while in the latter the oxidation of the pollutant occurs through radical chain reactions. On the opposite the electron is a strong reducing agent able to react with electron acceptor species, as is the case of dissolved oxygen to create superoxide radicals (O_2_^●−^) (reaction (3)), or other species with a redox potential more positive than that of the photocatalyst conduction band.

Photocatalysis initiation: semiconductor + *h*υ → e^−^ + h^+^ (1)

photo-oxidation: h^+^ + OH^−^ → OH^●^ or h^+^ + H_2_O → OH^●^ + H^+^ (2)

photo-reduction: e^−^ + O_2_ → O_2_^●−^ (3)

The efforts to optimize photocatalysts have been mainly focused on the increase of light efficiency, mainly by searching for solar-light-responsive photocatalysts and minimizing electron-hole recombination [60,63].

Among photocatalysts, nanosized semiconductor particles have met superior photocatalytic properties due to their large surface area, distinctly good dispersion capability and quantum size effects [63,69]. Yet, the large scale application of semiconductor nanoparticles has two major constrains: aggregation in suspension leads to fast loss of active sites, and consequent decreased photocatalytic efficiency, and the challenging separation, recovery, and reuse [63].

The synthesis of semiconductor hybrids and/or composites by coupling a semiconductor with organic/inorganic materials is a promising option to overcome the above mentioned drawbacks and challenges [70,71]. Usually, a hybrid material consists of a mixture of two or more phases at the nanometer scale or molecular level, and the hybrid material features do not correspond to the sum of the properties of the single phases but to synergistic interactions among them [70]. Typically, a composite consists of the dispersion of a micron-level phase or nanometer-size structures in a matrix, with the properties of the composite resulting from the addition of each phase contribution. To the best of our knowledge in photocatalysis most of times the terms hybrid and (nano)composite are indistinctively used to denote a material composed by two or more compounds and/or materials, regardless the establishment or not of specific chemical interactions between both phases, and the relative proportion of each one in the final mixture.

**Figure 2:**
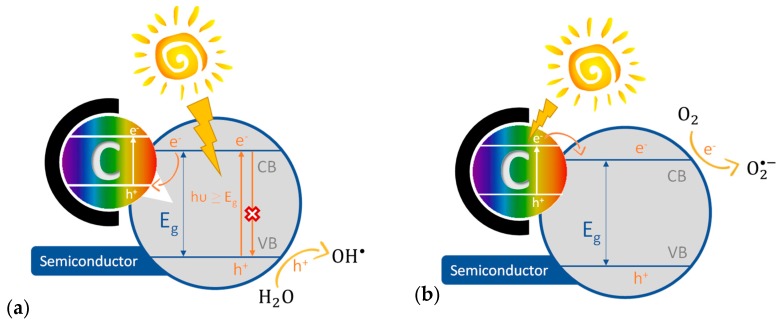
Simplified photocatalysis process schemes using semiconductor and carbon as addictive or support (**a**) charge separation in the presence of carbon, and (**b**) carbon acting as photosensitizer (inspired in [76,87]).

Due to their extensive surface area, black body properties, and high charge carrier mobility, carbon materials have been largely explored as dopants, hybridizing materials or supports of semiconductors. They are, undoubtedly, a very effective route to improve photocatalytic activity, as proved by the number of literature studies reviewing TiO_2_/carbon performance in the last decade [72,73,74,75,76,77,78,79,80,81,82,83,84,85]. For example Khalid et al. compared the performance of TiO_2_/carbon nanomaterials against bare TiO_2_ concluding that carbon doping and composites with activated carbons, fullerene, carbon nanotubes (CNTs), or graphene lead to materials with enhanced photocatalytic activity, adsorption capacity, electron scavenging and sensitization ability, extended visible light absorption, and also easier separation [73]. The enhanced photocatalytic activity due to the addition of carbon to a semiconductor can be reasoned in light of two mechanisms (i) improved charge separation in the presence of carbon, (ii) carbon acting as photosensitizer (Figure 2) [86]. When porous carbons are added to semiconductors, several synergistic effects due to adsorption must be considered. The charge separation mechanism by semiconductor/carbon composites was proposed by Woan et al. considering that carbon materials with metallic conductivity are able to trap the electron generated in the semiconductor, therefore hindering the recombination (Figure 2a) [76]. Wang et al. proposed the mechanism where the carbon material acts as photosensitized by transferring electrons to the conduction band of the semiconductor (Figure 2b) [87].

**Figure 3:**
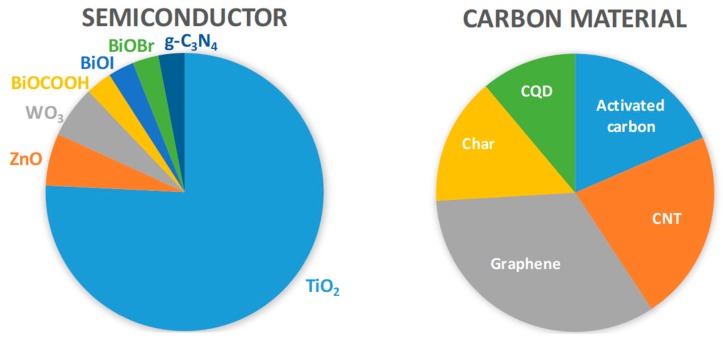
Semiconductor/carbon materials for the photodegradation of CBZ, DCF, and SMX according to the type of semiconductor (left) and type of carbon material (right). Source: ISI Web of Knowledge January 2019, search term “(carbamazepine OR diclofenac OR sulfamethoxazole) AND (photocat* OR photodegra*) AND (carbon)” search checked and refined by researcher.

In the following a review of the literature data (2010–2019) on the role of carbon in the performance of semiconductor/carbon materials for the enhanced photocatalytic degradation of CBZ, DCF, and SMX taking special attention to the improvement of the solar light harvesting will be presented. The distribution of literature studies on semiconductor/carbon materials for the photocatalytic degradation of each of these compounds according to the type of semiconductor and type of carbon material is presented in Figure 3. As expected TiO_2_ is by far the most studied semiconductor, counting 75% of the works but carbon materials are evenly distributed with graphene, CNTs and activated carbons representing, respectively, 33%, 22%, and 19% of the total contributions.

### 3.1. Semiconductor/C

Doping semiconductors is an option to improve their optical properties since the replacement of metal or nonmetal atoms by other elements, or the addition of atoms in interstitial positions, ideally maintaining the crystalline structure of the semiconductor, can shift the onset of the response from the UV to the visible region [88,89]. Several metals (e.g., W, V, Fe or Cu) and nonmetals (B, C, N, F, P, S, Cl, and Br) have been used to successfully dope TiO_2_ [88]. Among the nonmetals, N- and C-doping have similarities since both favor the formation of oxygen vacancies, enhancing the photocatalytic activity of TiO_2_ in the visible region [89]. The theoretical study by Valentin et al. reveals that at low C concentration the characteristics of the C-doped TiO_2_ depends on the O availability during the synthesis: substitution of O by C atoms and O vacancies are favored under O_2_-poor conditions, while interstitial C and substitution of Ti by C atoms is preferred at O_2_-rich conditions [89].

Irie et al. attributed the visible light responsivity of C-doped TiO_2_ (anatase), prepared by mild oxidation of TiC and further annealing under O_2_, to the 0.32% of C atoms located at O sites [62]. Albeit the very small carbon content in the doped TiO_2,_ it was enough to shift the absorbance edge and shoulder to the visible light region (bandgap narrowing). C-doped TiO_2_ (anatase) catalysts can be prepared by hydrolysis of TiCl_4_ with nitrogen bases (e.g., tetrabutylammonium hydroxide) followed by calcination at 400 °C, being proved that optical properties are dependent on the doping percentage [90]. Materials with 2.98% and 0.43% of C absorb light at 400–700 nm and were five times more active than an N-doped for the artificial solar light degradation of 4-chlorophenol, while the unmodified TiO_2_ was almost inactive [90]. TiO_2_/C-0.43% was also effective for the gas-phase photodegradation of acetaldehyde, benzene, and carbon monoxide in air under diffuse indoor light [90].

Recently C-doped semiconductors have also been successfully tested for the photocatalytic degradation of CBZ and DCF under UV and visible light (Table 3) and, as expected, TiO_2_ is by far the most explored semiconductor. The majority of the studies use initial DCF or CBZ concentrations in the mg/dm^3^ range, deionized water, and single-solute conditions.

In 2017, Surenjan and co-authors reported the effectiveness of C-doped spherical TiO_2_ in the anatase form for the photodegradation of DCF and CBZ after 4 h under visible light since after this time none of the pharmaceuticals or respective degradation intermediates and products were identified (mineralization degrees higher than 98%) [91]. The characterization of the doped photocatalyst reveals that C atoms may substitute Ti sites or occupy interstitial positions. The authors found that in the presence of glucose the degradation rate of CBZ decreases (from 0.0348 min^−1^ in single-solute to 0.0249 min^−1^) indicating that glucose acts as a sacrificial agent. In dual-solute conditions (i.e., DCF and CBZ) the rate of CBZ degradation has always a more pronounced decrease than that of DCF but even thought a complete removal of both PhCs was achieved in 210 min [91].

In a distinct approach, Sambandam et al. evaluated the effect of the morphology of C-doped TiO_2_ (anatase form) catalytic activity for the degradation of CBZ under visible light [92]. The authors proposed that in comparison with other crystal morphologies the rice grain shaped catalysts enhanced visible light degradation of CBZ (*k*_1_ of 0.094 min^−1^ vs. 0.059–0.02 min^−1^ for the other morphologies) due to the conjugation of co-exposed low energy/high energy {101}/{001} facets thermodynamically stable or not, and high surface area (229 m^2^/g vs. 33–62 m^2^/g for the other morphologies) [92].

**Figure 4:**
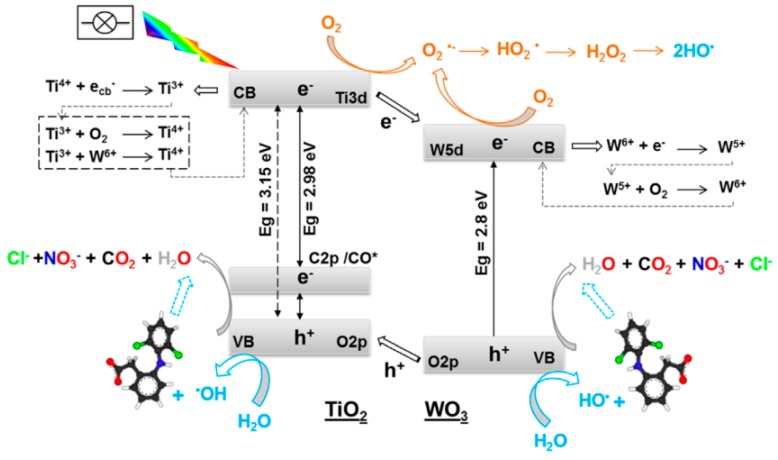
Mechanism of charge carrier separation proposed by Cordero-Garcia et al. [94] for photoexcited WO_3_/TiO_2_-C (Reprinted with permission [94], Copyright 2019, Elsevier).

The contribution of C-doping for enhanced semiconductor photodegradation of DCF under simulated solar light is clearly illustrated in the manuscript published by Cordero-Garcia et al. [94]. C-doping at percentages as low as 0.1–0.18 wt.% has a positive effect on: (i) the narrowing of the WO_3_/TiO_2_ band gap, (ii) the decrease of bandgap values to the visible light region (i.e., E_g_ values (in eV) of 3.15 for TiO_2_-0.04%C, 3.10 for WO_3_/TiO_2_-0.05%C, 3.05 for WO_3_/TiO_2_-0.1%C, and 2.98 for WO_3_/TiO_2_-0.18%C), and (iii) lower recombination e^−^/h^+^ [94]. The incremental slight displacement of the adsorption edge of the catalysts to the visible region proved to increase their mineralization efficiency for DCF, from 65% in the bare TiO_2_ to 76.2% in the WO_3_/TiO_2_, since WO_3_ acts as acceptor center for the photogenerated electrons. Values around 80% were attained for the C-doped mixed oxide (78.1% and 82.4% for, respectively WO_3_/TiO_2_-0.1%C and WO_3_/TiO_2_-0.18%C). Regardless the catalyst, the DCF degradation kinetic is always one order of magnitude faster than the TOC removal profile pointing out the formation of degradation products (possibly quinine imide compounds) more recalcitrant than parent DCF. The synergetic effect of C and WO_3_ on the properties of anatase under solar irradiation was explained by the authors according to the mechanism presented in Figure 4. The incorporation of C atoms created a narrow C2p band just above TiO_2_ O2p valence band allowing the visible light absorption and further transference of these photogenerated electrons to the Ti 3d conduction band. The dual-oxide W/Ti system allows the trapping of the photogenerated electrons improving the charge carrier separation. In both oxides, the holes can react with water to produce OH^●^ while the e^−^ can react with adsorbed O_2_ that is reduced to superoxide radicals and further reacts with protons to form HO_2_^●^ [94].

### 3.2. Semiconductor/Activated Carbon

As mentioned, the use of semiconductors has several drawbacks related namely with aggregation, high recombination of photogenerated e^−^/h^+^ pairs, limited activity under solar light and post-separation. To overcome these issues the scientific community explores different strategies. Back in early 1989, Tanguay et al. reported that anatase TiO_2_ supported on carbon felt was photoactive for the degradation of dicholomethane, and allowed easy separation of the catalyst from the reaction mixture [97]. Between 1998 and 2001, Matos et al. reported that the addition of an amorphous carbon phase (i.e., activated carbon) to semiconductor in mixed suspensions enhance the photoactivity for the degradation of organic pollutants [98,99,100]. The immobilization of semiconductor photocatalysts onto solid supports is an option to improve their performance, and among the several materials explored, activated carbons meet the criteria requested to be considered valuable supports since they (i) allow strong adherence between catalyst and support, (ii) the catalyst reactivity is not affected by the attachment process, (iii) have high specific surface area, and (iv) have a high adsorption affinity towards the pollutants [71]. In fact, several studies reveal that the use of activated carbon as support material can increase the photodegradation rate by progressively allowing an increasing quantity of substrate to come in contact with the immobilized metal oxide by means of adsorption [71], by minimizing the recombination e^−^/h^+^ of the metal oxide [101], and due to the black character of activated carbons harvesting the solar spectrum is favored. The studies aiming to evaluate the synergetic effects of semiconductor/carbon nanocomposites revealed that an increased adsorption of the contaminants onto the activated carbon phase, followed closely by a transfer through an interphase to the TiO_2_ phase, gives a complete photodegradation process [71,101]. In 2011, Lim et al. reviewed the synergistic adsorption-photocatalytic processes of TiO_2_/activated carbon composites addressing the challenges and further developments for water treatment and reclamation. Authors propose the coupling of these composites with membrane separation technologies to recover and regenerate the composites and also to enhance solar harvesting by using N-doped activated carbons [74]. The improvement of solar spectrum harvesting and catalysts immobilization strategies are also identified by Chong et al. as urgent needs in order to provide cost-effective photocatalytic technologies for water treatment sector [57].

Furthermore, as recently highlighted in the review on the photochemical activity of nanoporous carbons by Bandosz and Ania [102], activated carbons can play more than a secondary role in photochemical processes. Since 2010, works by Ania and co-workers have proven photoactivity of activated carbons due to their ability to absorb light and convert the photogenerated charge carriers into chemical reactions [103,104,105,106]. Velasco et al. identified the effect of nanoconfinement of the adsorbed aromatic species (phenol) in the pores of the nanoporous carbons as playing an important role in the photoactivity of these solids [102,103,104,105]. The optical properties of these materials are also linked with their heterogeneity (disorders/defects) along with the presence of C sp^2^ and sp^3^ configurations, and since their electrical conductivities, although being lower than that of graphene of carbon nanotubes, are higher than that of semiconductors contributing to lower the e^−^/h^+^ recombination [102].

The works reporting the use of semiconductor/AC composites for the photodegradation of CBZ, DCF, and SMX are listed in Table 4 and, again, TiO_2_ is the semiconductor tested and in only one study solar light irradiation is used.

Rioja et al. evaluated the effect of TiO_2_/powdered activated carbon (TiO_2_/PAC) composites’ synthesis method (physical, mechanical and chemical mixtures), and UV irradiation type on the competitive degradation of five PhCs, including CBZ, DCF, and SMX, in various water matrices (deionized, synthetic and spiked real water) [107]. The authors concluded that, in general, the presence of powdered activated carbon (PAC) improved the removal of PhCs due to the additive effect of adsorption and catalysis. It was also shown that the performance of the composites was dependent on the synthesis method and calcination temperature (overall removal): physical mixture (≈90%) > mechanical or chemical mixture calcined at 400 °C (70–80%) ≈ bare TiO_2_ (≈75%) > mechanical or chemical mixture calcined at 500 °C (≈60%). These variations were mainly due to SMX, and particularly, CBZ removal. The nature of the water matrix played a crucial role in the performance of the composites. For synthetic and spiked real water (tap water, river water, wastewater) photocatalysts deactivation was observed when compared with the assays performed in deionized water in similar conditions. However, the removals obtained in sea water were comparable to those of deionized water indicating that the high ionic strength appears not to compromise the photodegradation of the PhCs [107]. Interestingly, the CBZ removal and the two acidic PhCs (ibuprofen and clofibric acid) is more dependent on the water matrix. DCF and SMX present almost constant overall removal efficiencies regardless the presence/absence of PAC and the type of water matrix. In deionized water, DCF had the fastest photodegradation kinetic (0.1–0.4 min^−1^) while SMX presented the slowest one (0.01–0.03 min^−1^).

Ziegmann et al. found no photoactivity of two commercial PACs selected to degrade target PhCs (CBZ one of them) under UV light irradiation and reported the detrimental effect of the presence of PAC [108]. The authors considered that the high CBZ adsorption affinity for PAC, and the increase of the suspension turbidity due to the presence of the carbon particles, justify the low performance of the composite TiO_2_/PAC when compared with the bare TiO_2_. The initial removal of CBZ by adsorption was counterbalanced by a slower photodegradation rate and the values of dissolved organic carbon indicate that the rate of mineralization was much lower than the rate of degradation [108].

The enhancement of photoactivity through the immobilization of TiO_2_ onto AC was proved by Alalm et al. that studied the degradation of DCF, among other PhCs, using a solar reactor in a competitive scenario [109]. The removal efficiency for DCF was improved from 68% with bare TiO_2_ to 85% using TiO_2_/AC. Moreover, the composite also allowed total removals of amoxicillin and ampocilin. The photocatalytic degradation of the four PhCs in multi-solute conditions follows the Langmuir-Hinshelwood kinetic model. According to this mechanism, before degradation species must be adsorbed and this last process is faster for the TiO_2_/AC composite regardless the PhC. Higher removal rates with TiO_2_/AC were obtained in a large pH range (3–10), being the highest value observed for pH ≥ 7, which is, close to the values usually found in secondary effluent (wastewater). This is an advantage of this process in comparison with photo-Fenton (faster and complete degradation in 1–2 h) that is favored at pH 3, causing corrosion of mechanical facilities and the need for an extra neutralization step. Finally, according to the authors’ estimation, operating and total costs of using TiO_2_/AC solar photocatalysis were lower than those for bare TiO_2_ [109], thus highlighting the potential of these systems to be used in real water treatment facilities.

### 3.3. Semiconductor/CNT

Carbon nanotubes (CNTs) whose synthesis was reported by Iijima in 1991 [111] are another interesting class of carbon materials due to their outstanding structural, thermal and electronic properties associated with their one dimensional (1D) structure [73]. CNTs are classified as single-walled CNT (SWCNTs) and multi-walled CNT (MWCNTs) [111], presenting specific surface areas > 150 m^2^/g, excellent mechanical properties and their high electron mobility can be compared to those of metals, i.e., they may exhibit metallic conductivity [76]. As highlighted in several reviews published over the last decade, the combination of all these attributes makes CNT superior supports to enhance semiconductors photoactivity [72,73,76,78,85,112].

Woan et al. summarized the three mechanisms proposed in the literature to explain the enhancement of photoactivity in TiO_2_/CNT composites: (i) CNTs inhibit recombination, (ii) photosensitization due to e^−^/h^+^ pair generation in the CNTs, and (iii) CNTs act as impurities through Ti-O-C bonds [76]. In mixtures or nanocomposites semiconductor/CNT there are strong interfacial electronic effects between both players and the large electron-storage capacity of CNTs allows them to accept photon-excited electrons. As a consequence of the inherent electron density delocalization of the carbon matrix, the e^−^/h^+^ recombination is retarded or hindered [76,102]. MWCNT were reported to have self-photoactivity under visible light due to the presence of structural defects and vacancies [113], justifying a large number of studies using MWCNT instead of SWCNT [85]. The sensitizing role of MWCNT for visible light photoactivity was proposed by Wang et al. [87]: the electron photogenerated in the CNT is injected into the conduction band of the TiO_2_ and the positively charged CNT remove an e^−^ from the TiO_2_ valence band creating a h^+^, allowing the reaction with O_2_ (e^−^) and H_2_O (h^+^) to generate the oxygen reactive species (O_2_^●−^ and OH^●^, respectively). The third mechanism is more complex since two distinct contributions of TiO_2_/CNT are reported [76]. In the first one MWCNT act as carbon-doped TiO_2_ since the Ti-O-C bond extends the light absorption to longer wavelengths with potential to improve photoactivity and solar-light harvesting. The second is related to the electronic configuration of the CNTs that justifies the highest performance of CNTs prepared by arc-discharge CNT over CVD-grown ones, due to higher electrical conductivity and less defects of the former [76].

Owing to their great potential the use of semiconductor/CNT composites for the photodegradation of organic contaminants has been of considerable scientific interest and the works focused on the photodegradation of CBZ, DCF and SMX are listed in Table 5. As in the previous sections, TiO_2_ is the most tested semiconductor. Half of the works evaluated the performance of the composite under solar irradiation, and the majority use single-solute conditions and deionized water, not allowing the comparison of the behavior of the material, nor accounting for competition of other PhCs or organic matter.

**Figure 5:**
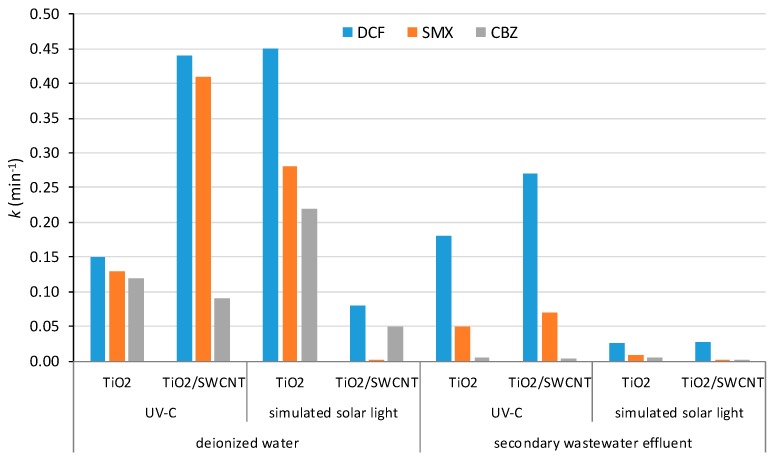
Effect of irradiation light and water matrix in first order kinetic constants (*k*) for DCF, SMX, and CBZ multi-solute photodegadation by TiO_2_ and TiO_2_/SWCNT (data from [114]).

In 2015 Murgolo et al. tested the multi-solute photocatalytic degradation of 22 PhCs, in the ppm concentration range, under UV-C and simulated solar light in deionized water and secondary wastewater effluent. The first-order constant rates for DCF, SMX, and CBZ are summarized in Figure 5 [114]. In the most favorable experimental conditions—UV irradiation and deionized water—for DCF and SMX the first-order constant rates obtained with composite TiO_2_/SWCNT are much higher than with TiO_2_ P25, while for CBZ TiO_2_ similar values were observed. For secondary wastewater effluent irradiated with UV-C light the photodegradation rates of DCF and SMX are also favored with the composite and a detrimental effect of the real wastewater is clearly shown. Regarding simulated solar light irradiation, the only interesting results were those obtained with TiO_2_ in ultrapure water. The photodegradation rate of the three PhCs addressed in this review follows the order DCF > SMX > CBZ [114]. So, in line with the high persistence of CBZ in the environment, due to the low photolysis profiles in water streams, according to these results CBZ is also more resistant to photodegradation regardless the irradiation source and water matrix. The reuse of TiO_2_/SWCNT was tested for five cycles with five PhCs, including SMX and CBZ, under UV light. No variation of rate constants were observed for all but ibuprofen. Lastly, it is important to stress that this work clearly demonstrates the importance of performing studies in real water matrices since the results obtained in synthetic solutions do not allow a direct translation to water treatment facilities.

Other studies listed in Table 5 systematically report enhanced visible light harvesting for the composites’ semiconductor/CNT when compared with the bare semiconductor [115,116,117,118,119].

Zhu et al. prepared composites WO_3_/MWCNT with increasing CNT contents verifying that higher CNT amount lower both the band gap energy and e^−^/h^+^ recombination. The composite WO_3_/MWCNT-4 (4 g of MWCNT) presented the best compromise between the band gap energy (2.52 eV), the dispersion in water and the amount of MWCNT [115]. SMX degradation efficiency under simulated solar light followed the order WO_3_ (25%) < WO_3_/MWCNT-2 (42%) < WO_3_/MWCNT-4 (65%) < WO_3_/MWCNT-8 (73%). The composite WO_3_/MWCNT-4 only lost 5% efficiency after four reuse cycles [115]. The mechanism proposed for the SMX photocatalytic degradation under visible light irradiation involves the transference of the photo excited electron from the WO_3_ conducting band to the CNT which acts as an electron trap to hinder e^−^/h^+^ recombination. The electron will react with O_2_ to produce O_2_^●−^, while the hole reacts with water and/or surface hydroxyl to generate OH^●^, these radicals will decompose the SMX [115].

Martinez et al. used TiO_2_/MWCNT_ox_ and TiO_2_ for the photocatalytic degradation of CBZ [116] and DCF [118] under UV-C and near UV-Vis irradiation (single-solute and deionized water). The TiO_2_/MWCNT_ox_ with a 10:1 weight ratio absorbes at higher wavelengths than TiO_2_ (P25, anatase or rutile) allowing improved sunlight harnessing [116,118]. The addition of 21% of O_2_ (V/V) contributes to a huge increase in the degradation rate of CBZ and DCF by P25 leading to half-life times lower than 5 min under near UV-vis light [116,118]. The faster degradation of the PhCs is attributed to the reaction between them and HO_2_^●^/O_2_^●−^, and/or to the strong electrophilic character of dissolved O_2_ which may reduce unfavorable e^−^/h^+^ recombination. Albeit the improved sunlight harvesting, compared with anatase for UV irradiation or P25 for near UV-vis irradiation, the composite has worst photoactivity for CBZ and DCF degradation (UV-C or near UV-vis), possibly due to OH^●^ inhibition in the presence of MWCNTs. The fact that CBZ and DCF are electron-rich molecules that may transfer e^−^ to MWCNT conducting band may also justify the slower oxidation process [116,118].

Czech & Buda tested nanocomposites TiO_2_-SiO_2_/MWCNT with 0.01% to 17.8% of CNT against TiO_2_-SiO_2_ for the photocatalytic degradation of CBZ [117] and DCF [119]. The addition of SiO_2_ promotes the dispersion of TiO_2_ [119] while the addition of MWCNT steadily reduces the band-gap of the composite TiO_2_-SiO_2_/MWCNT from 3.2 eV to 2.2 eV when the CNT content increases from 0.15% to 17.8%, shifting absorption to visible [117]. CNT acts as a dopant for up to 3.5 wt.% CNT but as support for higher concentrations. Under UV-A irradiation TiO_2_-SiO_2_/MWCNT with 17.8 wt.% of CNT allows faster CBZ degradation, through a decomposition pathway distinct from that obtained by P25, and the formation of degradation products with low toxicity to *D. magma* and *V. fisheri*. Both composites with TiO_2_-SiO_2_/MWCNT with 0.01 wt.% of CNT was effective than bare P25 for the adsorption and degradation of the DCF under UV-A or solar light. Most probably due to their small weight percentage in composites, MWCNTs mainly contributes to the degradation step [119]. However SiO_2_-TiO_2_ presented even higher photocatalytic activity than those doped with MWCNT, being more active under visible light than under UV-A and allowing a decrease of bioluminiscent inhibition of *V. fisheri* (from > 90% to values ≈ 20%) [119]. It must be also highlighted that MWCNTs are effective solar-responsive metal-free photocatalysts for DCF degradation regardless the light source (UV-A and visible). In fact MWCNTs removed ≈ 50% DCF by adsorption plus more 30–40% by photocatalytic degradation [119].

### 3.4. Semiconductor/(r)GO

Graphene is a two-dimensional (2D) sheet of carbon atoms connected by sp^2^ bonds with an aromatic π electron system [120], it has a high thermal conductivity (≈5000 W/(m × K)), allows excellent mobility of charge carriers at room temperature (2000 cm^2^/(V × s)), high optical transmittance (≈97.7%), and has an extremely high theoretical specific surface area (≈2600 m^2^/g) [121,122]. The first report of graphene synthesis and properties dates from 2004 by Novosevol et al. and since then graphene has been extensively explored by the scientific community in several applications, and it also caught the attention of industry.

Graphene is the precursor of graphene oxide (GO) and reduced graphene oxide (rGO), both showing high efficiency for water treatment due to their surface functionalities (e.g., hydroxyl, carboxyl and epoxy) [72]. Due to the exceptional features of graphene and derived nanomaterials, these solids are attractive for many applications mainly in environmental protection and energy production/storage. The use of graphene-based semiconductor photocatalysts has been a hot research topic since graphene joins the most promising properties of CNTs and activated carbons, i.e., the high electron mobility with high pore structure and adsorption capacity. The advances in graphene-based semiconductor photocatalysis have been addressed in several review papers [72,73,80,81,82,83,84,121,122,123].

The enhanced photoactivity of semiconductor/graphene composites can be understood in light of the mechanisms previously described for semiconductor and activated carbon or CNT composites. The advantages and shortcomings of the use of activated carbons, CNTs and graphene on the semiconductor composites were systematized by Awfa et al. [72] identifying that easy recovery from reaction media follows the trend activated carbon > CNT > graphene, while the overall process cost follows the opposite trend. High mechanical strength and improved thermal stability, large electron storage capacity, and superior metallic conductivity are some of the advantages of adding CNT and graphene to semiconductors. Due to graphene’s 2D structure, the interfacial contact between graphene and the semiconductor usually provides higher photoactivity [72]. The more difficult separation of the semiconductor/graphene composites can be overcome during the synthesis to obtain, for example, 3D aerogels [124], composites immobilized onto a solid supports [125] or adding magnetic features [126]. As reviewed by Dong et al. [127] the enhanced visible light photocatalytic activity of semiconductor/graphene composites is attributed to (i) the improved e^−^/h^+^ mobility and suppression of the photo-generated e^−^/h^+^ recombination, (ii) to their higher surface adsorption capacity for organic molecules through π-π interactions, and (iii) full and intimate contact through chemical bonds of the metal semiconductor with carbon and oxygen atoms.

Certainly due to their potential graphene-based materials were the most representative group of composites studied for the photocatalytic degradation of CBZ, DCF, and SMX. It must be also noted that the majority of studies listed in Table 6, were tested under under visible irradiation, and only two works evaluate the performance of the photocatalysts in real water. In all but one study TiO_2_ was the semiconductor selected.

Lin et al. synthesized TiO_2_/rGO with rGO weight percentages 0.1–10% coated and tested the photoactivity of the composites for the photocatalytic degradation of three PhC, including SMX and CBZ, under UV and visible light [125]. The best performing photocatalyst was TiO_2_/rGO-2.7% due to (i) lower band gap energy, (ii) formation of a heterojunction interface leading to improved charge separation [125]. Under UV-vis irradiation SMX removal is more effective than CBZ (92% vs. 54%) but both PhCs attain similar mineralization degrees (54–59%) indicating that although SMX presents faster degradation its oxidation intermediates need similar time as those of CBZ to achieve mineralization [125]. The immobilization of the composite onto optical fibers proved to be a promising strategy since after 15 cycles (i.e., corresponding to 45 h) under UV-vis irradiation the ibuprofen removal efficiency by TiO_2_/rGO-2.7% was over 80% [125].

SMX photocatalytic degradation by TiO_2_/rGO composites under visible light was also tested by Yang et al. demonstrating that TiO_2_/rGO is an activator of persulfate for SMX and other PhCs degradation through SO_4_^●−^ and OH^●^ [129]. Karaolia et al. studied SMX adsorption and photocatalytic degradation in spiked real urban wastewater effluent verifying that bare P25 is more efficient that TiO_2_/rGO prepared photocatalitically or by hydrothermal synthesis [128].

Three studies report CBZ photochemical degradation using single-solute conditions and UV irradiation. The best performing TiO_2_/graphene composite attained higher CBZ degradation than bare TiO_2_ [124,126,130] and adding magnetic properties [126] or 3D graphene structure [124] allowed several reuse cycles. Appavoo et al. [130] and Nawaz et al. [124] concluded that there is an optimum percentage of GO or rGO to assure the best compromise between CBZ adsorption and photocatalytic degradation. Appavoo et al. report mineralization degrees of 80% when composites with 1% and 2% of GO are used, while P25 only reaches 64%.

Metals or peroxodisulfate were used as addictive to improve photocatalytic degradation of DCF with semiconductor/graphene composites under visible light [131,132,133]. Cheng et al. conclude that both Pd and rGO in the TiO_2_ nanotubes/Pd-rGO contribute to the improved light harvesting and effective separation of the photogenerated charge carriers, and identify OH^●^ as the most relevant radicals for DCF degradation [131]. Li et al. reported 100% DCF removal and 55.8% mineralization when irradiating Ag-BiOI/rGO under visible light during 80 min, maintaining the performance after three reuse cycles [132]. The authors propose that Ag enhance the separation e^−^/h^+^ while the rGO with extended π-electron conjugations has strong charge transfer capability and is the potential sink of e-. Moreover, rGO can function as e- acceptor, and transfer due to its excellent charge carrier conductivity [132]. The work by Chen et al. followed a more applied perspective by evaluating the effect of the water matrix and solution pH in the DCF degradation by TiO_2_/rGO in the presence of peroxodisulfate under visible light [133]. The authors verified that the increase of solution pH has a detrimental effect on DCF degradation rate and environmental factors also interfere in the degradation. The presence of Cl^−^ facilitates DCF degradation but the presence of HCO_3_^−^ as well as tap water, lake water, river water matrices decrease DCF degradation. In this photodegradation system both O_2_^●−^ and h^+^ play significant roles in DCF degradation, peroxodisulfate behaved like an electron acceptor to enhance e^−^/h^+^ separation and generation of additional reactive oxygen species, and rGO served as electric conductor, so both addictive increase photocatalytic efficiency of the composite for DCF degradation [133].

### 3.5. Semiconductor/Carbon Quantum Dots (CQDs)

Carbon quantum dots (CQDs) were firstly reported by Xu et al. [134] in 2004 just a few months earlier than graphene, but these fluorescent carbon nanoparticles only received their most common name – CQD – from Sun et al. in 2006 [135]. CQDs are approximately flat or quasi-spherical carbon nanoparticles (< 10 nm) composed by amorphous to nanocrystalline cores with predominantly sp^2^ carbon (i.e., graphitic carbon and/or graphene and GO sheets) fused by diamond-like sp^3^ hybridized carbon intersections [136,137]. CQDs have excellent aqueous solubility due to the considerable amounts of carboxylic surface moieties, presenting oxygen content between 5 and 50% [135,136,137]. CQDs have demonstrated capability of harnessing long wavelength light and their ability to exchange energy with solution species offers excellent opportunities for their use as photocatalysts [136]. Furthermore, CQDs present up-conversion fluorescent emission, an optical phenomenon wherein the fluorescence emission wavelength is shorter than the used excitation wavelength. This property is particularly important for in vivo bioimaging since near IR (NIR) region is usually preferred [136] but is also a very valuable feature to improve semiconductor photoactivity under full sunlight spectrum [137]. In fact, CQDs proved to be excellent additives in solar photocatalytic systems since their ability to harness the full spectrum of sunlight and up-convert visible light into shorter wavelength light triggers the semiconductor charge separation (i.e., e^−^/h^+^ generation and hinders recombination) which by reaction with H_2_O/O_2_ will produce the active oxygen species needed to promote contaminants degradation [136,137].

Regarding the target PhCs, to the best of our knowledge, so far semiconductor/CQD composites were only explored for the photodegradation of DCF (Table 7). The three very recent studies explore the solar light photoactivity of the composites in single-solute conditions, and only one evaluates the influence of distinct water matrices.

Chen et al. report the superior performance of BiOCOOH/CQD composites under visible-light for the degradation of DCF when compared with P25 [138]. The incorporation of CQDs allows the composite to absorb in the visible range presenting band gaps 3.42–2.84 eV, as the percentages of CQDs increase from 1 to 5%. The best photocatalytic activity is obtained for BiOCOOH/CQD-2% (98% DCF removal vs. 51.5% for BiOCOOH and only 15% for P25), allowing to attain 90% DCF even after four reuse cycles. This behavior was mainly attributed to the generation of O_2_^●^^−^ and holes [138]. It is also remarkable that in just 2 h this composite effectively mineralized, detoxify and dechlorinated DCF, and it also presented high performance for the degradation of the antibiotic sulfadimidine (>90%) and rhodamine B (>75%), proving its potential for application in water remediation strategies. Authors propose CQDs act as photosensitizers of the BiOCOOH, with the electron transfer properties of the CQDs being beneficial to constrain the e^−^/h^+^ recombination and their up-conversion properties contributing to enhancing the visible photoactivity of the semiconductor [138].

In a more sustainable approach, Liu et al. tested metal free composites g-C_3_N_4_/CQD for the visible light photodegradation of DCF [140]. Once again, the introduction of optimized amounts of CQDs improved visible light harvesting and increased e^−^/h^+^ separation. Composite g-C_3_N_4_/CQD-0.05 showed the fastest total DCF removal just 1 h irradiation and degrades 90% of the DCF after five reuse cycles [140]. Alkaline medium favors a faster DCF degradation. O_2_^●−^ plays the dominant role in the DCF degradation process which is not dependent on the holes, which allowed the proposal of a photosensitation-like mechanism. After 1.5 h of irradiation of g-C_3_N_4_/CQD-0.05, the DCF mineralization reached 54% and although, in general, the intermediates are less toxic than the parent compound they are still classified as very toxic, pointing out the need of longer irradiation times [140].

In 2017, Song et al. used g-C_3_N_4_ for the photodegradation of sulfonamides, including SMX, under visible light attaining 90% removal under distinct water quality parameters [141]. SMX was degraded by O_2_^●−^ and holes, and the degradation pathways were proposed.

Wang et al. compare the efficacy of composites of TiO_2_ nanosheets with two distinct surface energy facets or P25 with N-doped CQD for DCF degradation under broad spectrum irradiation [139]. The results reveal that TiO_2_ facets play a major role in the photodegradation process with the superior performance of TiO_2_{001}/N-CQD. This behavior was attributed to the synergistic effect of the high oxidation activity of exposed {001} facets, up-conversion properties of the N-CQDs and efficient charge separation in the direct Z-scheme heterojunction [139]. Under visible light, composites with TiO_2_{101} and TiO_2_{001} photodegrade, respectively, around 80% and more than 90% of DCF after 1 h of irradiation, while composite with P25 and the three bare TiO_2_ samples degraded less than 20% of DCF [139]. Despite the high DCF removal by TiO_2_{001}/N-CQD, after 1h of visible light irradiation, only 50% mineralization was achieved. After four reuse cycles, the composite effectively degrades 80% of DCF. Contrarily to what was reported by Liu et al. [140] for g-C_3_N_4_/CQD, with this composites the increase of solution pH from 3 to 11 has a detrimental effect on the DCF degradation rate (from 0.09 to 0.01 min^−1^) [139]. The influence of the water matrix was studied showing that the presence of transition metals and humic acids restrain the photodegradation of DCF. On the contrary the assays in natural waters (river water, sea water, and wastewater effluent) show that photocatalytic degradation of DCF is only slightly inhibited (84.0–84.6% vs. 90.9%, in deionized water) [139].

### 3.6. Semiconductor/Char

Chars are typically obtained by the carbonization of biomass through the removal of other elements than carbon, and transformation of the precursor in an amorphous carbon-rich material usually named biochars [142]. Chars can also be designated as pyrochars, hydrochars or acid-chars depending on the carbonization process, i.e., pyrolysis, hydrothermal, or acid-mediated carbonization, respectively [142,143]. Chars have incipient pore networks and depending on the precursor and synthesis procedure (i.e., carbonization method, temperature, and time) it is possible to tailor their surface chemistry from highly acidic to basic surface chemistries rich in oxygen groups or even other electron rich atoms (e.g., nitrogen, sulfur, or phosphorus) in order to meet with the needs of a specific process. The oldest known use of char (carbonized wood, coal, or partially devolatilized coals), also known as charcoal, dates to the Stone Age when the material was used as black pigment for cave painting [144]. Greeks and Romans used charcoal in the treatment of various diseases, and Hindus for water treatment. In the nineteenth century, it played a crucial role in sugar refining, and as gas filters in London sewage ventilation systems [142]. Recently Chen et al. compared the photoactivity of a pyrochar vs. a hydrochar, obtained from the same precursor, verifying that due to the abundant photoactive oxygenated surface groups, the hydrochar could generate more reactive species under sunlight thus enhancing the degradation of the target pollutant (sulfadimidine) [143].

Along with the carbon materials previously discussed, biochars have also been tested to enhance semiconductor activity. The works focused on SMX and CBZ photodegradation under UV or visible light irradiation are gathered in Table 8. All the studies were performed in single-solute conditions and in one case the photocatalytic activity of the semiconductor/char composites in deionized water and spiked river water were compared.

Xie and co-workers reported 80% SMX removal after 3 h under simulated visible light using a Zn-TiO_2_/biochar composite [145]. This composite outperformed TiO_2_/biochar and bare TiO_2_, which was explained considering that Zn-doping effectively reduced the agglomeration of TiO_2_ and decrease the crystal size. On the other hand, the higher photocurrent of Zn-TiO_2_/biochar justifies the higher charge transfer and separation [145]. The photoactivity of Zn-TiO_2_/biochar slightly decreases after the first cycle but remains almost constant in the next four reuses. Water matrix has a crucial role in the composite performance: in spiked river water only 53.83% SMX removal, the presence of SO_4_^2−^, Cl^−^, and NO_3_^−^ also has an inhibitory effect possibly due to trapping of OH^●^ or capture of h^+^ to hinder production of OH^●^ [145].

CBZ photodagradation was studied by Li et al. with magnetic Fe_3_O_4_/BiOBr/biochar, Fe_3_O_4_/BiOBr, and bare BiOBr [146]. At pH 6 Fe_3_O_4_/BiOBr/biochar (10% biochar, 0.05 g Fe_3_O_4_) is the best performing photocatalyst: highest rate constant (0.01777 min^−1^), CBZ removal similar to that of BiOBr (95%), 70% mineralization (only ≈ 30% for BiOBr, ≈ 55% for Fe_3_O_4_/BiOBr). The species OH^●^, h^+^ and O_2_^•−^ seem to take part in the CBZ photodegradation, suggesting that oxygen radicals play the most important role. After five reuse cycles under visible irradiation, Fe_3_O_4_/BiOBr/biochar degraded 90% of the CBZ, and in the fourth cycle more than 60% mineralization is achieved. Like in the previous study the presence of Cl^−^ and SO_4_^2−^ has detrimental effect on CBZ degradation. Humic acid has opposite effects since while when they are present at low concentration photodegradation decreases but at higher concentrations they behaved as photosensitizers promoting CBZ photodegradation [146].

Khraisheh et al. assayed TiO_2_/biochar, with coconut shell powder char, for the CBZ photodegradation under UV light irradiation in the presence of O_2_ flow [147,148]. Regardless of the biochar amount, all composites presented higher adsorption and photocatalytic activity than bare TiO_2_, biochar and granular activated carbon (GAC). For pH 3 to 11 the degradation rate of CBZ is almost constant (0.05 min^−1^) attaining 89.2–94.4% CBZ removal [148]. Interestingly after 1h of UV-C irradiation GAC and composite attain, respectively, ≈90% and ≈99% CBZ removal while bare TiO_2_ and biochar only attain 42% of CBZ removal [148]. While the 90% removal with GAC is mainly attributed to adsorption (nanoporous solid), in the case of the biochar, that has an incipient pore structure, the 42% CBZ removal may result from the combined adsorption and photocatalytic degradation. Regarding recyclability TiO_2_/biochar also has better performance since after 11 reuse cycles it removes 60% CBZ while for the other solids the removal is <25% [148]. The enhanced removal of CBZ with the TiO_2_/biochar composite is attributed to the surface area that allows adsorbing CBZ that is further photodegraded by the reactive oxygen species produced by the semiconductor [148].

### 3.7. Overview of the Degradation Pathways and Intermediates/Products

One of the major challenges of photocatalytic degradation of PhCs is to achieve total mineralization. Although in optimized conditions high and fast degradation of the target pollutants can be attained, the mineralization rate is usually slower than the removal rate leading to the presence of degradation intermediates and/or products whose toxicity can be higher than that of the parent compound.

**Figure 6:**
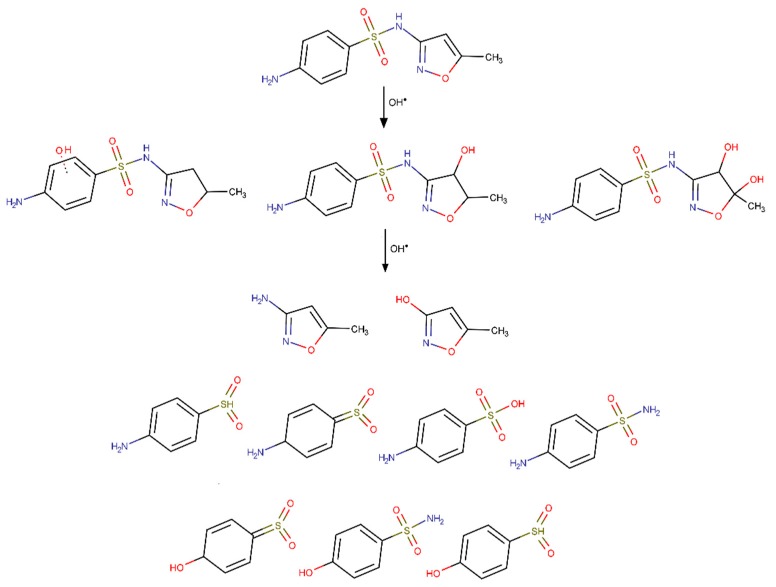
SMX degradation pathways and intermediates by WO_3_/CNT and/or Zn-TiO_2_/biochar under simulated solar light irradiation (based on [115,145]).

Among all the works focused on SMX photocatalytic degradation by semiconductor/carbon materials only two address the mechanism and propose decomposition pathways [115,145], in both cases under solar light irradiation. Despite the use of distinct composites—WO_3_/MWCNT and Zn-TiO_2_/biochar—four common transformation intermediates/products were identified and similar degradation pathways are proposed (Figure 6): (i) hydroxylation (OH^●^ attack) on the benzene ring or isoxazole ring of SMX generating mono- or di-hydroxyl derivatives, and (ii) then OH^●^ cleavages the S-N bond. [115,145]. Zhu et al. propose that continuous radical attack to the benzene and isoxazole rings leads to bond cleavage [115]. The degradation products identified present m/z down to 99, being mainly substituted aromatic compounds. However, none of the studies report the mineralization degree or evaluated the toxicity of the solution after photocatalysis.

**Figure 7:**
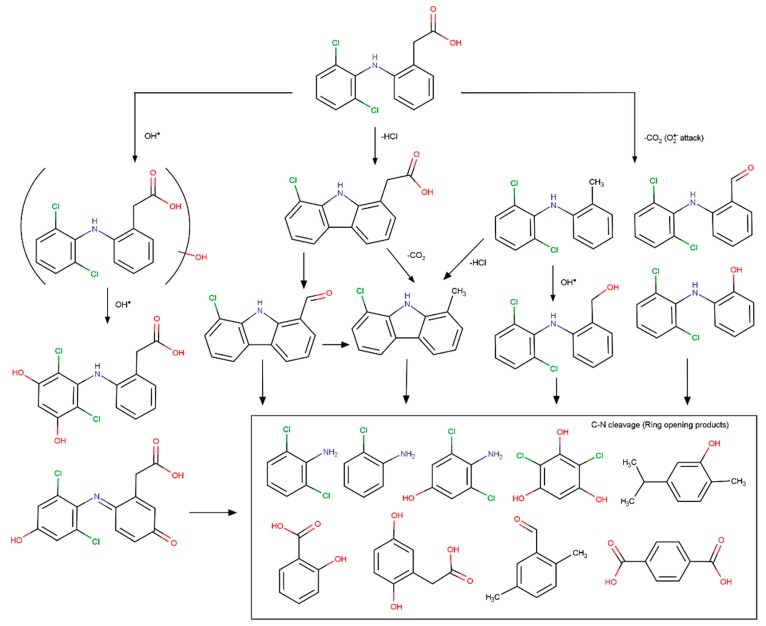
DCF degradation pathways and intermediates by Ag-BiOI/rGO, TiO_2_/rGO with peroxodisulfate, TiO_2_{001}/N-CQD, g-C_3_N_4_/CQDs, and/or BiOCOOH/CQDs under visible light irradiation. The OH substituents which can appear in different positions in the rings cross the parenthesis mark (based on [132,133,138,139,140]).

The visible-light driven photodegradation pathways of DCF by semiconductor/carbon materials were proposed by different research groups that explored the effect of CQDs and rGO addition to various semiconductors (i.e., TiO_2_, TiO_2_{001}, BiOCOOH, Ag-BiOI and g-C_3_N_4_) [132,133,138,139,140]. Hydroxylation, decarboxylation (through O_2_^●−^ attack) and dechlorination, followed by C-N bond cleavage are the transformation pathways generally proposed (Figure 7). The smallest degradation products identified are single–ring aromatic compounds with chlorine, hydroxyl, carboxyl, and amine substituents (m/z from 127 to 194). By using g-C_3_N_4_/CQD Liu et al. attained DCF mineralization degrees of 54% after 1.5 h of visible light irradiation and estimated the toxicity of DCF and its degradation intermediates/products by quantitative structure-activity relationship (QSAR) prediction [140]. The authors conclude that, according to acute toxicity values, although in general the degradation intermediates are less toxic than parent DFC they are still classified as very toxic (LD_50_ for rat is 244 mg/kg). However the photocatalytic process with g-C_3_N_4_/CQD significantly decreased the bioaccumulation factor and development toxicity, compared to parent DCF. Moreover while DCF is “mutagenic positive” only one of the degradation intermediates (obtained by chlorine removal and ring closure) has the same classification, and the remaining are “mutagenic negative” [140]. Czech et al. verified that after photodegradation treatment of DCF solution with SiO_2_-TiO_2_/MWCNT, and regardless the use of UV-A or visible light, the *V. fisheri* bioluminescence inhibition decreased to values around 20% (i.e., in the border between toxic and non-toxic classification) while the toxicity of the initial DCF solution was higher than 90% [119]. The photodegradation of DCF with BiOCOOH/CQD composites under visible light irradiation demonstrates that treatment time is a critical factor [138]. After 40–50 min of treatment DCF was totally removed, but the mineralization degree was only 47.9%, certainly due to the abundant degradation intermediates. The assays with *V. fisheri*, *D. magna*, and *D. subspicatus* systematically reveal that the toxicity of the degradation intermediates presents a maximum at 40-50 min of treatment (more than double of the parent DCF) [138]. However, after 2 h of treatment DCF was effectively mineralized, detoxified and dechlorinated, clearly demonstrating the need of prolonged treatment times to assure higher mineralization degrees and consequently lower toxicity.

**Figure 8:**
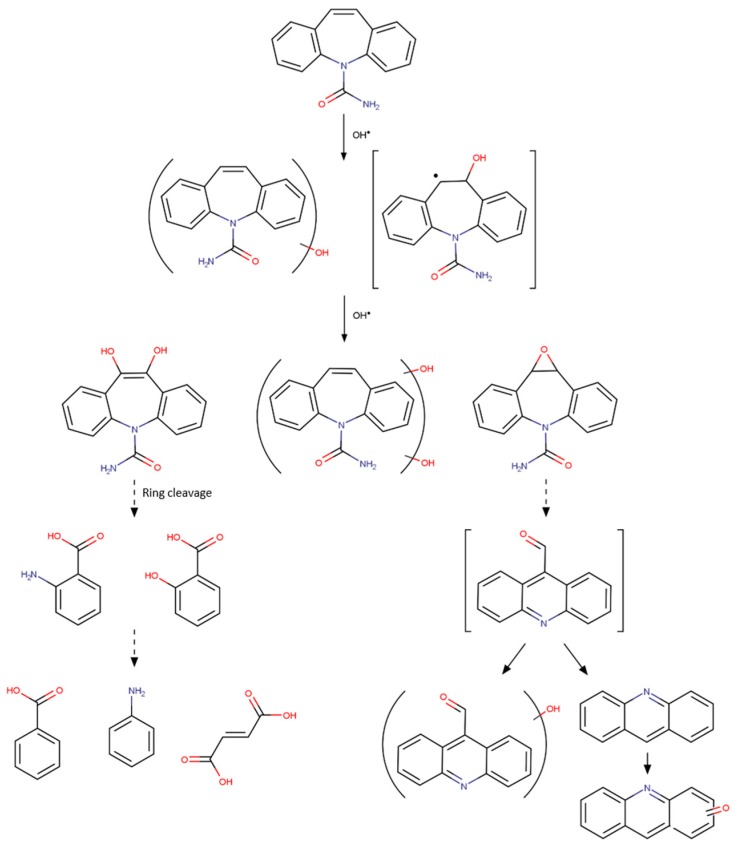
CBZ degradation pathways and intermediates by TiO_2_/rGO, TiO_2_/MWCNTox and/or Fe_3_O_4_/BiOBr/biochar under distinct irradiation conditions (UV, near UV-vis, and Visible LED light). Short-lived intermediates presented between [brackets] while those between (parenthesis) correspond to stable intermediates, the OH substituents which can appear in different positions in the rings cross the parenthesis mark (based on [116,124,146]).

Regarding CBZ three of the four studies reporting degradation pathways and products used TiO_2_ derived composites with MWCNT, rGO, and biochar [116,124,146]. Figure 8 resumes the CBZ degradation pathways and intermediates by TiO_2_/rGO, TiO_2_/MWCNTox and/or Fe_3_O_4_/BiOBr/biochar under distinct irradiation conditions (UV, near UV-vis, and Visible LED light). All the proposed degradation pathways start with the hydroxylation of the aromatic system or the olefinic double bond, and two of the studies reported that the hydroxylated intermediates suffer ring cleavage to originate substituted single-ring aromatic compounds (m/z from 93 to 137) or even an aliphatic dicarboxylic acid (*m*/*z* of 115). The study reporting the use of Fe_2_O_3_/BiOBr/biochar for the degradation of CBZ under visible light attained 70% mineralization indicating that most of the degradation intermediates could be eliminated to small organic molecules [146]. The toxicity of model wastewater containing CBZ before and after photodegradation treatment with TiO_2_/SiO_2_/MWCNTox under UV light was evaluated by Czech et al. that reported lower toxicity of the treated solution to *V. fisheri* and *D. magna* when compared with the non-treated wastewater [117].

Overall, regardless of the target PhC—SMX, DCF, and CBZ—and the composite used for photocatalytic degradation, the main products are mainly substituted single-ring aromatic compounds. The degradations pathways of SMZ and CBZ start with the hydroxylation of the aromatic moieties. DCF has the most complex degradation pathway since it can start by hydroxylation, decarboxylation and/or dechlorination. Besides OH^●^ attack to the aromatic systems, O_2_^●−^ plays a prominent role in the decomposition of the DCF carboxylic acid group. Despite the high number of degradation pathways, DCF is the PhCs with a larger amount of intermediates and products identified.

## 4. Summary and Outlook

The versatility of carbon element is well expr essed in this review, the combination of C atoms with semiconductors (C-doping) or the use of carbon materials—activated carbons, CNT, graphene, and derived materials, CQD and biochars—as supports or doping materials in composites with semiconductors allows to enhance photocatalytic activity and sunlight harvesting. The black body character of the carbon materials associated with properties as distinct as texture, conductivity, or quantum effects are certain parameters responsible for the improved performance of the composites.

Regarding the three target pharmaceuticals of this review, DCF was by the most explored in photocatalytic degradation studies with semiconductor/carbon material (19 papers vs. 8 papers for SMX and 14 papers for CBZ), most likely due to its presence between 2013 and 2018 in the Watch Lists published by European Commission. The interest of the academy towards CBZ probably results from its high recalcitrant behavior in conventional wastewater treatment and high persistence in the environment that also supports the proposal of this pharmaceutical as a possible anthropogenic marker. In fact, also the few studies that evaluate the photocatalytic degradation of more than one of the three targets PhCs systematically identify CBZ as the PhC most resistant to photocatalytic degradation followed by DCF and SMX, in line with photolysis data and persistence after conventional wastewater treatment (biodegradation). Therefore, it is important to continue developing enhanced technologies to effectively restrain the dissemination of these recalcitrant PhCs, with special focus on CBZ, in the environment.

Regarding photocatalytic degradation of these PhCs, only few studies are focused on real water matrices, with the catalytic performance being mainly evaluated using deionized water solution at pH not close to the values of real water. However, the studies reporting data obtained in real water matrices reveal that the high performance of the catalyst in deionized water is not a guaranty for good results in spiked wastewater.

The majority of studies reviewed are focused on evaluating the removal efficiency of novel catalysts and composites for a specific contaminant, in most cases not assessing the mineralization rate. However the data available clearly demonstrates that the mineralization process demands longer operation times than the degradation/removal of the target PhC. Moreover, when toxicological assays are reported the data reveal that the transformation intermediates formed during the initial degradation process may be as toxic as, or even more toxic than, the pristine PhC.

As a final remark it must be stressed that regardless of the type of carbon material present in the composite, the literature data is almost unanimous to demonstrate the beneficial effect of the carbon doping or addition for the sunlight harvesting and better overall performance of the semiconductor/carbon catalysts. The enhanced photocatalytic activity of the semiconductor/carbon composites is mainly attributed to the electron transfer properties of the carbon material and better charge separation. However, there are also reports where the carbon material (CQDs) act as photosensitizers. The bibliometric analysis shows the great interest of the scientific community in the more conducting carbon materials—CNT and graphene-derived materials—and also the recent growing research in CQD. Nevertheless, more traditional carbon forms, as is the case of nanoporous carbons or biochars, may be more sustainable and feasible for large scale applications not only due to the production cost but mainly due to the toxicity of the crystalline carbon forms. In fact, in vivo studies reveal the health issues associated with CNT and graphene-derived materials due to their rigid structures that damage the cell walls [149,150]. Another advantage of nanoporous carbons is related to their well-known pore structure that, besides contributing to the overall removal of the target pollutant, may also allow the adsorption of the degradation intermediates and products thus contributing to overall decrease of the toxicity level.

Lastly, it must also be mentioned that self-photoactivity of some carbon materials for these recalcitrant PhCs, in both UV and solar irradiation, was observed in more than one work, point out the need to further explore the potential of metal-free carbon materials for the photocatalytic degradation of priority contaminants, ideally under sunlight irradiation and in real water matrices.

## Figures and Tables

**Table 1:** Target pharmaceutical compounds molecular structures, therapeutic classes and properties.

Pharmaceutical Molecular Structure	Therapeutic Class	Properties
Carbamazepine (CBZ) 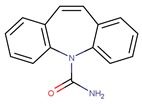	Antiepileptic	MW = 236.3 g/mol p*K*_a_ = 13.9 [4] Log *K*_ow_ = 2.45 [4] Water solubility (25 °C) = 18 mg/dm^3^ [4] *k_H_* = 1.1 × 10^−10^ atm m^3^/mol [4] *K*_d,prim_ < 0.020 L/g_ss_ [5] *K*_d,sec_ = 0.0012 ± 0.0005 L/g_ss_ [5] *k*_biol_ ≤ 0.01 L/g_ss_·d [6] *K*_OH_ = 8.8 × 10^9^ ± 1.2 × 10^9^ 1/M s [7]
Diclofenac (DCF) 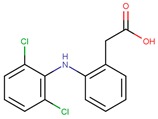	AnalgesicAnti-inflammatory	MW (acid) = 296.1 g/mol MW (sodium salt) = 318.1 g/mol p*K*_a_ = 4.15 (acid) [8] Log *K*_ow_ = 4.51 [8] Water solubility (25 °C) = 2.37 mg/dm^3^ [8] *k_H_* = 4.73 × 10^−12^ atm m^3^/mol [8] *K*_d,prim_ = 0.495 ± 0.032 L/g_ss_ [5] *K*_d,sec_ = 0.016 ± 0.003 L/g_ss_ [5] *k*_biol_ ≤ 0.1 L/g_ss_·d [6] *K*_OH_ = 7.5 × 10^9^ ± 1.5 × 10^9^ 1/M s [7]
Sulfamethoxazole (SMX) 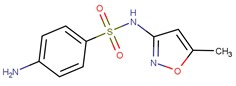	Antibiotic	MW = 253.3 g/mol p*K*_a1_ = 1.6, p*K*_a2_ = 5.7 [9] Log *K*_ow_ = 0.89 [9] Water solubility (37 °C) = 610 mg/dm^3^ [9] *k_H_* = 6.4 × 10^−13^ atm m^3^/mol [9] *K*_d_ = 0.256 ± 0.169 L/g_ss_ [10] *k*_biol_ ≤ 0.2 L/g_ss_·d [6] *K*_OH_ = 5.5 × 10^9^ ± 7 × 10^9^ 1/M s [7]

p*K*_a_, negative log of acidity constant(s), *K*_ow_, octanol-water partition coefficient, *K*_d_, sorption constant on activated sludge (*K*_d,prim_ and *K*_d,sec_ for, respectively, the primary and secondary treatment), *k*_biol_, pseudo first-order degradation rate constant (1 g SS^−1^ day^−1^), *K*_OH_, second-order rate constant with OH^●^ radicals.

**Table 2:** Early detections of carbamazepine (CBZ), diclofenac (DCF), and sulfamethoxazole (SMX) in wastewater and environmental water.

PhC	Type of Water	Concentration (μg/L)	Publication Year	Ref.
CBZ	STP effluents	3.7 (max 6.3)	1998	[19]
	Rivers and streams	0.82 (max 1.1)		
	Ground water	Up to 0.900	2001	[26]
	STP influent (Frankfurt/Mains)	2.2	2001	[18]
	STP effluent (Frankfurt/Mains)	2.0		
	Ground water	Up to 1.1		
	Drinking water	Up to 0.030		
	STP influent (Berlin)	1.78 (max 3.80)	2002	[17]
	STP effluent (Berlin)	1.63 (max 5.00)		
	Surface water (Berlin)	0.4–1.1		
	Surface water (Berlin)	0.025–1.075	2002	[24]
	STP effluent (France)	0.98–1.2	2003	[23]
	STP effluent (Greece)	1.03		
	STP effluent (Italy)	0.3–0.5		
	STP effluent (Sweden)	0.87		
DCF	Sedimentation tank effluent	≤2	1996	[27] (in [21])
	River Rhine	0.015–0.304		
	Different rivers	0.038–0.489		
	STP effluents	1.6 (max 2.1)	1998	[19]
	Rivers and streams	0.80 (max 1.2)		
	Influent Swiss STP	0.47–1.92	1998	[22]
	Effluent Swiss STP	0.31–0.93		
	Swiss lakes/rivers	0.001–0.370		
	Brazilian STP	0.4 (max ≈ 1.4)	1999	[20]
	River water	0.02–0.06		
	STP influent (Frankfurt/Mains)	1.9	2001	[18]
	STP effluent (Frankfurt/Mains)	0.58		
	Ground water	0.93		
	Drinking water	Up to 0.006		
	Ground water	Up to 0.590	2001	[26]
	STP influent (Berlin)	3.02 (max 7.10)	2002	[17]
	STP effluent (Berlin)	2.51 (max 4.70)		
	Surface water	< 0.1–0.6		
	Ground water (Berlin)	Up to 0.38		
	Surface water (Berlin)	Up to 1.030	2002	[24]
	STP effluent (France)	0.25–0.41	2003	[23]
	STP effluent (Greece)	0.89		
	STP effluent (Italy)	0.47–5.45		
SMX	River water	≈1	1983	[28] (in [21])
	STP effluents	0.9 (max 2.0)	1999	[25]
	Surface water	0.14 (max 0.48)		
	Ground water	Up to 0.47		
	Ground water	Up to 0.410	2001	[26]
	STPs effluent (Berlin)	0.9	2002	[17]
	STP effluent (France)	0.07–0.09	2003	[23]
	STP effluent (Greece)	0.09		
	STP effluent (Italy)	0.01–0.03		
	STP effluent (Sweden)	0.02		

**Table 3:** Photocatalytic degradation of CBZ and DCF by C-doped semiconductors.

PhC	Catalyst	Experimental Conditions	Concluding Remarks	Ref.
CBZ DCF	TiO_2_/C	Microwave synthesis: spherical catalyst (anatase) of 50 nm [TiO_2_/C] = 50 - 350 mg/dm^3^ [PhC]_0_ = 50 - 250 µg/dm^3^ (each PhC) Continuous O_2_ pH 6.5 – 8.5 Visible (intensity 4000–10,000 lx) 60 min dark (ads) + 4 h light (deg) Single-solute and multi-solute Deionized water	250 mg/dm^3^ of catalyst allow higher and faster degradation High [PhC] lowers degradation efficiency, likely due to the higher competition of PhCs and intermediates for active sites Optimum light intensity: 8000 lx pH 7.5 allows higher degradation rate of CBZ and DCF At pH > 7.5 repulsion between the photocatalyst, negative surface charge and excess of OH^−^ may prevent the formation of OH^●^ In the presence of glucose CBZ degradation rate decreases Dual-solute (DCF and CBZ) degradation profiles: DCF removal was faster (0.0334 min^−1^) while CBZ removal rate slightly decreases compared with single-solute assay (0.0348 min^−1^ vs. 0.0247 min^−1^) After 4 h irradiation no CBZ or DCF is detected, > 98% TOC removal at higher [PhC]_0_ (5 mg/dm^3^) and catalyst loading (500 mg/dm^3^)	[91]
CBZ	TiO_2_/C	Microwave synthesis: anatase TiO_2_/C catalyst (50–70 nm) with controlled shape [TiO_2_/C] = 230 mg/dm^3^ [CBZ]_0_ = 50 µg/dm^3^ Continuous O_2_ flow 300 mL/min Visible (150 W, >400 nm, 7700 lux) 45 min dark (ads) + 2 h light (deg) Single-solute & deionized water	Mainly graphitic carbon is deposited on the surface of TiO_2_ From the four TiO_2_/C morphologies the rice grain shaped nanocrystal catalysts presented enhanced visible light degradation of CBZ CBZ photodegradation obeys the pseudo-first order kinetic model for all morphologies with *k*_1_ (in min^−1^) following the sequence of 0.094 (rice grain) >> 0.059 (spherical) > 0.044 (distorted spherical) > 0.020 (hexagonal)	[92]
DCF	TiO_2_/C TiO_2_/C,N	Sol-gel synthesis: mesoporous TiO_2_ (with Amberlite and calcination at 300 and 550 ºC) [DCF]_0_ = 10–50 mg/dm^3^ UV-C (1.5–2.2 mW/cm^2^) 24 h in dark (ads) + 2 h light (deg) Single-solute & model wastewater Continuous flow reactor	TiO_2_/C (pure anatase) present the best photocatalytic performance attaining ≈ 80% DCF degradation, ≈80% mineralization, and pseudo-first order rate constants of 1.0964 × 10^−2^ min^−1^ and 1.3242 × 10^−2^ min^−1^ High photoactivity of TiO_2_/C attributed to increased rates of e^−^ transfer with increasing specific surface area.	[93]
DCF	WO_3_/TiO_2_-C WO_3_/TiO_2_ TiO_2_	Sol-gel method: under acidic conditions (≈2 wt.% WO_3_, 0.1 or 0.18 wt.% C) [Catalyst] = 1 g/dm^3^ [DCF]_0_ = 10 mg/dm^3^ pH ≈ 7 Simulated solar light (Xe lamp 1500 W) > 290 nm Single-solute	C-doping narrows WO_3_/TiO_2_ band gap and decreases *E*_g_ All catalysts attain total DCF degradation after 250 kJ m^−2^ of accumulated energy but mineralization is favored at higher C-doping During degradation, pH decreases due to the formation of carboxylic acids and Cl^−^ release DCF degradation kinetic faster than mineralization process (10^−2^ vs. 10^−3^ min^−1^): intermediate products more recalcitrant than parent DCF Synergetic effects among semiconductors and C allows high catalytic activity of WO_3_/TiO_2_-C under solar light (changes in electronic structure)	[94]
DCF	ZnO/C ZnO TiO_2_	Composites obtained by high-thermal processing (900 °C) [Catalyst] = 0.5 g/dm^3^ (TiO_2_ – Aeroxide P25) [DCF]_0_ = 10 mg/dm^3^ UV-A 30 min dark (ads) + 1 h light (deg) Single-solute	ZnO/C has the highest efficiency (60% DCF degradation in 20 min) TOC removal profile similar to that of DCF indicating that TOC decrease is connected with direct DCF decomposition, 35% TOC remaining corresponds to DCF and transformation products DCF photodegradation follows pseudo-first order kinetic model: *k*_1_ (ZnO/C) = 8 *k*_1_ (P25) and *k*_1_ (ZnO/C) = 2.5 *k*_1_ (ZnO) Defects in ZnO structure of ZnO/C contribute to better DCF removal	[95]
DCF	TiO_2_/C	C-doped anatase TiO_2_ coated on glass plate (12.5% C) [DCF]_0_ = 0.5 mg/dm^3^ pH 6.2 – 7.2 UV-A (3 lamps 15 W, max 365 nm) Single-solute & deionized water	TiO_2_/C adsorbed UV-A light and also a range of visible light due to its smaller bandgap compared with bare TiO_2_ OH^●^ were generated and DCF was degraded (< quantification limit)	[96]

ads–adsorption, deg-degradation

**Table 4:** Photocatalytic degradation of CBZ, DCF, and SMX by semiconductor/activated carbon (PAC—powdered activated carbon, ACfiber—activated carbon fiber).

PhC	Catalyst	Experimental Conditions	Concluding Remarks	Ref.
SMX DCF CBZ	TiO_2_/PAC TiO_2_	Composites: physical, mechanical and chemical mixtures [TiO_2_] = 0.5 g/dm^3^ (Aeroxide P25) [PAC] = 2.5–35 mg/dm^3^ (commercial) [PhC]_0_ = 0.5 mg/dm^3^ (each PhC) UV-A (1.0 mW/cm^2^), UV-B (2.5 mW/cm^2^), UV-C (3.65 mW/cm^2^) 60 min dark (ads) + 30 min light (deg) Multi-solute (5 PhCs) Deionized water, synthetic matrix and spiked real water (tap water, river water, sea water, wastewater)	In deionized and river water UV-C allows higher overall removal Photocatalyst deactivation in synthetic and spiked real waters (mainly with HCO_3_^−^ and in wastewater effluent) In spiked sea water, ads+deg yields comparable to deionized water In deionized water physical mixture attains higher overall efficiency (~90%) than mechanical or chemical mixture composites (60–80%) In deionized water, degradation by TiO_2_ and TiO_2_/PAC mixture fits the first-order kinetic model: fastest for DCF and slowest for SMX Regardless of the type of water, PAC enhances overall efficiency and individual removal yield DCF 100% removal independent of PAC addition Cumulated kinetic constants (after dark) are worse than single PhC but still fit first-order model: 0.069 min^−1^ in deionized water, 0.070 min^−1^ in seawater, but only 0.029 min^−1^ in wastewater	[107]
CBZ	TiO_2_/PAC TiO_2_ PAC	1:5 wt. ratio TiO_2_:PAC (conglomerates in suspension) pH 7.5 (phosphate buffer) [TiO_2_] = 0.1 g/dm^3^ (Aeroxide P25) [PAC] = 0 - 20 mg/dm^3^ (commercial - Norit) [PhC]_0_ = 8 - 9 mg/dm^3^ (each PhC) UV light (Hg lamp) Single-solute & deionized water	Steam activated PAC (steam-PAC) originated higher amount of mixed agglomerates with TiO_2_ in suspension: tested in a hybrid process Steam-PAC did not allow CBZ degradation, neither did the other 3 PhCs Steam-PAC addition seemed to inhibit the rate of CBZ degradation: initial adsorption (17 to 59%) led to lower degradation rate (*i.e*., 0.009-0.012 min^−1^ (TiO_2_+PAC) vs. 0.022 min^−1^ (TiO_2_)) High CBZ affinity for PAC may decrease mobility, preventing the contact between CBZ and TiO_2_ resulting in a lower degradation rate. Turbidity increase due to PAC addition can also justify the low performance of the TiO_2_/PAC mixture. For a PhC with lower adsorption affinity (iomeprol), the detrimental effect of turbidity was compensated by the synergistic effect TiO_2_/PAC	[108]
DCF	TiO_2_/AC TiO_2_	TiO_2_:AC wt. ratio 2:1 and impregnation at 200 ºC [TiO_2_/AC] = 0.4 – 1.6 g/dm^3^ (AC – Adwic, TiO_2_ – Acros, commercial) [TiO_2_] = 0.2 – 0.8 g/dm^3^ [PhC]_0_ = 50 mg/dm^3^, 4 dm^3^ pH 3 - 10 Solar reactor with compound parabolic collectors PhCs mixture continuous circulated in a closed cycle 30 min dark (ads) + 3 h light (deg) Multi-solute (4 PhCs)	TiO_2_: Negligible PhCs adsorption (including DCF), after 90 min of irradiation 58% DCF removal attained, after further 90 min 68% DFC removal achieved. Faster PhCs degradation during the first 90 min attributed to OH^●^ radicals abundance, the slower degradation after 90 min attributed to catalyst deactivation Depending on the solution pH and PhC, composite TiO_2_/AC adsorbs 5–25% of the PhCs and attains 80 – 84% DCF removal at pH 5, 7, or 10 For 0.8 g/dm^3^ of catalyst loading, TiO_2_/AC attains ≥ degradation efficiency for the four PhCs and also contributes to a faster photocatalytic process than bare TiO_2_ despite the smaller amount of semiconductor PhCs photocatalytic degradation follows a Langmuir-Hinshelwood kinetic model: faster for the TiO_2_/AC composite regardless of the PhC Regardless of the PhC, TiO_2_/AC attain higher removal efficiency than bare TiO_2_ (51–85% vs. 43–75% for DCF)	[109]
DCF	Fe_3_O_4_/Ti_x_O_y_/ACfiber Fe_3_O_4_/Ti_x_O_y_ TiO_2_	Composite obtained by ultrasound irradiation [Catalyst] = 1.5 g/dm^3^ TiO_2_ synthesized and commercial [DCF]_0_ = 4.7 mg/dm^3^ UV light 2.5h light (adsorption+catalysis) Single-solute	Composites with and without ACfiber prepared by ultrasonic irradiation present the high removal of DCF (96% and 91%, respectively)	[110]

ads–adsorption, deg-degradation

**Table 5:** Photocatalytic degradation of CBZ, DCF, and SMX by semiconductor/CNT (SWCNT—single-walled carbon nanotubes, MWCNT—multi-walled carbon nanotubes).

PhC	Catalyst	Experimental Conditions	Concluding Remarks	Ref.
SMX DCF CBZ	TiO_2_/SWCNT TiO_2_	0.58 mg TiO_2_/mg SWCNT [TiO_2_] = [TiO_2_/SWCNT] = 0.1 mg/dm^3^ (TiO_2_: Aeroxide P25 and synthetic rod-like nanocristals) [PhC]_0_ = 0.2 – 0.5 mg/dm^3^ UV-C (0.10 W/cm^2^) and simulated solar light 320 – 700 nm (0.10 W/cm^2^) 30 min in dark (ads) Multi-solute (22 PhCs) Deionized water and real water (secondary wastewater effluent)	PhC mixture photodegradation follows the Langmuir-Hinshelwood mechanism showing first-order kinetic model In ultrapure water under UV light TiO_2_/SWCNT is more effective than TiO_2_ for DCF, SMX, and more 4 PhC, for the remaining 16 PhCs, (including CBZ) TiO_2_ is comparable or slightly better In real wastewater effluent under UV light, lower degradation rates compared with values in ultrapure water in similar conditions TiO_2_/SWCNT and TiO_2_ performance dependent on water matrix, irradiation, and PhC 5 reuse cycles with 5 PhCs (including CBZ and SMX) under UV light TiO_2_/SWCNT advantages: easy separation and reuse	[114]
SMX	WO_3_/MWCNT WO_3_	Hydrothermal synthesis: 400 mg Na_2_WO_4_·2H_2_O for 2, 4 or 8 mg MWCNT [WO_3_] = [WO_3_/MWCNT] = 0.5 g/dm^3^ (Commercial MWCNT) [SMX]_0_ = 10 mg/dm^3^ Simulated solar light 420 – 630 nm 1 h dark (ads) + 3 h light (deg) Single-solute & deionized water	CNT content increase in the WO_3_/MWCNT composites enhances visible light absorption (red-shift) Band gaps: 2.80 eV (WO_3_), 2.65 eV (WO_3_/MWCNT-2), 2.52 eV (WO_3_/MWCNT-4) and 2.32 eV (WO_3_/MWCNT-8) Similar photoluminescence emission spectra for bare WO_3_ and composites, but composites have lower e^−^/h^+^ recombination and charge separation is improved as the CNT content increases Removal under solar irradiation (3 h): WO_3_ (25%) < composites (42–73%) WO_3_/MWCNT-4 composite is the smarter choice The higher the WO_3_/MWCNT-4 dose (0.25 - 2.00 g/dm^3^) the better the SMX removal efficiency (40–88%) After 4 reuse cycles, WO_3_/MWCNT-4 only lost ~5% removal efficiency All radicals contribute to the SMX degradation with WO_3_/MWCNT-4 under solar light, being OH^●^ and O_2_^●−^ the most important ones Intermediates identified and 4 processes proposed for SMX photocatalytic degradation by WO_3_/MWCNT-4	[115]
CBZ	TiO_2_/MWCNT_ox_ TiO_2_ ZnO	Mixture and sol-gel synthesis:10:1 titania to MWCNT_ox_ [TiO_2_] = [TiO_2_/MWCNT_ox_] = 0.1-2 g/dm^3^, optimum 0.5 g/dm^3^ (TiO_2_ - Aeroxide P25 and lab-made (anatase and rutile), commercial MWCNT_ox_) [ZnO] = 0.5 g/dm^3^ (com. Evonik) [CBZ]_0_ = 8 mg/dm^3^ pH ≈ 6 (natural CBZ solution pH) 200 cm^3^/min O_2_/Ar (0-100 vol.% of O_2_, optimum 50 vol.% of O_2_) [H_2_O_2_] = 0–10 mM, optimum 5 mM UV-C and Near UV-vis (NUV-vis) 30 min dark (ads) + 1 h light (deg) Single-solute & deionized water	Composite TiO_2_/MWCNT_ox_ absorbs at higher wavelengths than TiO_2_: advantage considering sun-light harnessing Photolysis and photocatalytic degradation of CBZ upon UV-C irradiation, under NUV-vis light CBZ photolysis is negligible compared with photocatalysis Addition of O_2_ favors faster CBZ photodegradation UV irradiation: (1) addition of MWCNT does not promote CBZ photocatalytic degradation (pure photochemical process) (2) TiO_2_ efficiency: anatase > P25 > rutile NUV-vis irradiation (photocatalytic regime): (1) positive synergy for P25 and synthetic TiO_2_ with 70% anatase and 30% rutile (2) CBZ photocatalytic degradation trend TiO_2_ P25 > mixture TiO_2_ + MWCNT >>> TiO_2_/MWCNT_ox_+H_2_O_2_ 5 mM > TiO_2_/MWCNT_ox_ (3) ZnO degradation of CBZ 17% higher than with TiO_2_ P25 2 pathways proposed for CBZ photodegradation	[116]
CBZ	TiO_2_-SiO_2_/MWCNT TiO_2_	Sol-gel synthesis: composites with 0.15 – 17.8 wt.% CNT [Catalyst] = 0.5 g/dm^3^ [PhC]_0_ = 10 mg/dm^3^ (each PhC) TiO_2_ Aeroxide P25 for comparison UV (1.0 mW/cm^2^) 30 min dark (ads) + 2 h light (deg) Single-solute	Composites with anatase TiO_2_ (7–8 nm): E_g_ reduces from 3.2 eV to 2.2 eV as CNT content increases and visible light harvest improved Up to 3.5 wt.% CNT act as a dopant in TiO_2_/SiO_2_, for higher CNT content TiO_2_/SiO_2_ crystals are supported on the outer CNT surface Faster degradation with the composite presenting 17.8 wt.% of CNT (Pseudo-first order kinetic constant 0.0131 – 0.0743 min^−1^) Distinct decomposition pathway with TiO_2_ P25 and composites Addition of CNT during TiO_2_/SiO_2_ synthesis: enhances TiO_2_ activity, changes CBZ degradation mechanism, and transformation products in model wastewater have low toxicity to *D. magna* and *V. fisheri*	[117]
DCF	TiO_2_/MWCNT_ox_ TiO_2_	Same catalyst ref. [116] [TiO_2_] = [TiO_2_/MWCNT_ox_] = 0.1-2 g/dm^3^, optimum 1 g/dm^3^ [DCF]_0_ = 8 mg/dm^3^ pH ≈ 6 (natural DCF pH) 200 cm^3^/min O_2_/Ar [H_2_O_2_] = 0–5 mM, optimum 5 mM UV-C and Near UV-vis (NUV-vis) 30 min dark (ads) + 1 h light (deg) Single-solute & deionized water	Addition of O_2_ favors faster DCF photodegradation Complete photolytic and photocatalytic degradation of DCF upon UV-C and NUV-vis light after 30 min irradiation UV irradiation: DCF degradation trend anatase > rutile > TiO_2_/MWCNT_ox_ > no catalyst > TiO_2_ P25 NUV-vis irradiation (photolytic + photocatalytic regime): DCF photocatalytic degradation trend TiO_2_ P25 > anatase > no catalyst > TiO_2_/MWCNTox > rutile Considering the 8 identified intermediates the photocatalytic degradation of DCF is proposed	[118]
DCF	SiO_2_-TiO_2_/MWCNT SiO_2_-TiO_2_ TiO_2_	Sol-gel method: (0.01 wt.% MWCNT) basic and acid conditions, calcined in air (400 ºC) [Catalyst] = 0.5 g/dm^3^ TiO_2_ lab-made and P25 (Evonik) [DCF]_0_ = 10 mg/dm^3^ UV-A and visible light 30 min dark (ads) + 1 h light (deg) Single-solute	All prepared catalysts were more effective than TiO_2_ P25 for the adsorption+degradation of DCF under UV-A and solar light Regardless of the light source, MWCNT alone removes ≈ 50% DCF by adsorption and ≈ 30% more by photocatalytic degradation In composites, MWCNT mainly contributes to degradation SiO_2_-TiO_2_ presented higher photocatalytic activity than catalysts doped with MWCNT and are more active under visible light than under UV-A After treatment with SiO_2_-TiO_2_ and regardless of the light source the bioluminiscent inhibition of *V. fisheri* decreased to values ≈ 20% while the toxicity of the solution before treatment was > 90%	[119]

ads–adsorption, deg-degradation

**Table 6:** Photocatalytic degradation of CBZ, DCF, and SMX by semiconductor/graphene (GO—graphene oxide, rGO—reduced graphene oxide).

PhC	Catalyst	Experimental Conditions	Concluding Remarks	Ref.
SMX CBZ	TiO_2_/rGO TiO_2_	TiO_2_/rGO (0.1–10 wt.% GO:TiO_2_) coated on optical fibers Catalyst: 30 fibers of 10 cm coated with composite and TiO_2_ (synthesized and Aeroxide P25) [PhC]_0_ = 5 mg/dm^3^ (each PhC) pH 6 UV-vis (UV-B, UV-A and visible) 3 h dark (ads) + 3 h light (deg) Single-solute & deionized water	Without catalyst: CBZ removal is negligible while SMX decreases 30% Photoctalytic activity: synthesized TiO_2_ < TiO_2_ P25 < TiO_2_/rGO-2.7% (> 50% CBZ and > 90% SMX removals) While SMX removal is more effective than of CBZ (> 90% vs. ≈ 40%) both reach similar mineralization (54 - 59%) after 3 h irradiation: faster SMX degradation but intermediates need similar time as those of CBZ to achieve mineralization TiO_2_/rGO-2.7% durability assessed for ibuprofen during 15 cycles (45 h total contact time) under UV-vis irradiation: >80% removal	[125]
SMX	TiO_2_/rGO TiO_2_	*Ex-situ* methods: hydrothermal or photocatalytic treatment of TiO_2_ and exfoliated GO [Catalyst] = 0.1 g/dm^3^ (Commercial TiO_2_, Aeroxide P25) [PhC]_0_ = 0.1 mg/dm^3^ pH 5.2-6.2 Simulated visible light (63 W/m^2^) 30 min dark (ads) + 1 h light (deg) Multi-solute (3 antibiotics, bacteria *E. coli*, genomic DNA content, and antibotic resistant encoding genes) Spiked urban wastewater effluents	Composite prepared by photocatalytic treatment has the highest adsorption (≈30%) but the best overall (adsorption + photodegradation) performance achieved with P25 (87% vs. 50% and 15% for, respectively, photocatalytical and hydrothermal composites) For other 2 antibiotics (clarithromycin and erythromycin) the best performing photocatalyst is the photocatalytic-derived composite	[128]
SMX	TiO_2_/rGO TiO_2_	Hydrothermal synthesis [Catalyst] = 1 g/dm^3^ [Persulfate] = 20 mmol/dm^3^ [PhC]_0_ = 10 mg/dm^3^ Visible light (300 W Xe lamp, > 420 nm, 2000 W/m^2^) 30 min dark (ads) + 1 h light (deg) Single-solute & deionized water	TiO_2_/rGO is an efficient activator of persulfate for visible light SMX degradation: 52% SMX degradation (0.055 min^−1^) and 26% mineralization Enhanced visible light harvesting and efficient charge separation seem to provide TiO_2_/rGO more photo-induced e^−^ for persulfate activation Both SO_4_^●−^ and OH^●^ contribute to SMX photodegradation	[129]
CBZ	TiO_2_/Fe_3_O_4_/rGO TiO_2_	[Catalyst] = 0.1 mg/cm^3^ [CBZ]_0_ = 1.18 μg/dm^3^ TiO_2_ commercial (Aeroxide P25) UV-C (0.8 W/cm^2^) 30 min dark (ads) + 1 h light (deg) Single-solute Deionized water	TiO_2_ allows 95% removal of CBZ while the composite attained 97%. Similar pseudo-first order kinetic constant for TiO_2_ and composite (5.4–5.5 × 10^−2^ min^−1^) Easy recover and reuse due to magnetic properties	[126]
CBZ	TiO_2_/GO TiO_2_	Microwave hydrothermal method: 1 – 10% GO and 0.2 mg TiO_2_ P25, Aeroxide [TiO_2_/GO] = 10 mg/dm^3^ [CBZ]_0_ = 0.3 mg/dm^3^ UV-A (1.8 mW/cm^2^) 30 min dark (ads) + 5 - 20 min light (deg) Single-solute & deionized water	2% GO in the composite allows the faster CBZdegradation, 1% and 1.5% allow higher pseudo-first order kinetic rates than P25. Higher amounts of GO have a detrimental effect on photodegradation. Composites containing between 1% and 2% of GO reach 80% mineralization while P25 only attains 64% TOC reduction Higher performance of composites TiO_2_/GO is attributed to the transfer and transport of e^−^ from TiO_2_ conducting band to the graphene sheets lowering the e^−^/h^+^ recombination	[130]
CBZ	TiO_2_/3D rGO TiO_2_	Physical mixture or hydrothermal synthesis (1:1 to 4:1 wt.% ratio TiO_2_/GO, with L-ascorbic acid) [Catalyst] = 0.5 g/dm^3^ (TiO_2_ lab-made and commercial) [CBZ]_0_ = 10 mg/dm^3^ UV-A (13.5 W/m^2^) 40 min dark (ads) + 1.5 h light (deg) Single-solute	The composite prepared *ex-situ* (direct use of TiO_2_ nanoparticles) with 2:1 wt.% ratio TiO_2_/rGO presented the best performance (≈99% CBZ degradation, 0.0473 min^−1^ pseudo-first-order rate constant) Synergistic effect of the chemically bonded rGO and TiO_2_ in the composite (0.0265–0.0473 min^−1^) supported by a lower efficiency of physically mixed system (0.0037 min^−1^) worse than TiO_2_ (0.0067 min^−1^) Composite 2:1 has a consistent photoactivity during 5 reuse cycles Photodegradation mechanism of CBZ proposed and degradation products identified	[124]
DCF	TiO_2_ nanotubes/Pd-rGO	Photoelectrode prepared by electro-deposition [DCF]_0_ = 5 mg/dm^3^ (each PhC) Visible light (35 W Xe lamp) 2 h dark (ads) + 12 h light (electrodegradation) Single-solute	The TiO_2_ nanotubes/Pd-rGO photoelectrode has enhanced photocurrent density and charge carrier concentration, attaining 58.4% for DCF degradation under visible light Pd and rGO improve light harvesting and effective charge separation OH^●^ radicals are the major reactive specie in the DCF photocatalytic removal, to a lesser extend O_2_^●^^−^, H_2_O_2_ and h^+^ are also identified After 5 reuse cycles, the DCF degradation was almost constant	[131]
DCF	Ag-BiOI/rGO BiOI Ag-BiOI BiOI/rGO	Hydrothermal synthesis [Catalyst] = 1 g/dm^3^ [DCF]_0_ = 10 µg/dm^3^ Visible light (300 W) 30 min dark (ads) + 2 h light (deg) Single solute	Ag-BiOI/rGO is the best performing catalyst (0.026 min^−1^), attaining 100% DCF removal and 55.8% mineralization in 80 min (visible light) After 3 reuse cycles, Ag-BiOI/rGO removes 100% of DCF in 2 h Ag enhance the separation e^−^/h^+^ while the rGO is the potential sink of e^−^ and can accept and transfer e^−^ (excellent charge carrier conductivity) DCF intermediates identified and two degradation routes proposed	[132]
DCF	TiO_2_/rGO	Hydrothermal method: wt. ratios TiO_2_:rGO 100:0.1, 100:0.5 and 100:1 [Catalyst] = 0.3 g/dm^3^ [Peroxodisulfate(PDS)] = 0–5 mM [DCF]_0_ = 4 mg/dm^3^ pH 4–9 Visible (blue) light LED (450–455 nm, ~3.84 mW/cm^2^) 12 min dark (ads) + 20 min light (deg) Single-solute Deionized water, tap water, lake water, and river water	Without PDS TiO_2_/rGO removes 45% DCF while TiO_2_ attains ≈25% With PDS TiO_2_/rGO (0.5–1%) degrades 90% DCF (0.106–0.109 min^−1^) after 20 min of irradiation pH increase from 4 to 9 has a detrimental effect on DCF degradation (0.201 min^−1^ to 0.0453 min^−1^) Environmental factors affect the degradation of DCF PSD is an e^−^ acceptor to enhance e^−^/h^+^ separation and generation of additional reactive oxygen species, rGO served as an electric conductor Both O_2_^●−^ and h^+^ play significant roles in DCF degradation Total removal of DCF and 65% mineralization after 25 min irradiation, 1.5 h needed to reach ≈90% mineralization 12 intermediates identified and 3 possible degradation pathways proposed Composite lost only ≈10% efficiency after four reuse cycles	[133]

ads–adsorption, deg-degradation

**Table 7:** Photocatalytic degradation of DCF by semiconductor/CQD.

PhC	Catalyst	Experimental Conditions	Concluding Remarks	Ref.
DCF	BiOCOOH/CQD TiO_2_	Composites obtained by ultrasonic dispersion (3h at 180 °C) [Catalyst] = 0.6 g/dm^3^ (TiO_2_–Aeroxide P25) [PhC]_0_ = 4 mg/dm^3^ pH 7 Kinetic, by-product and toxicity: Visible light (350 W Xe lamp, 1.15 mW/cm^2^) with 420 nm cut-off Photocatalyst mechanism tests: UV-A (390–400 nm, 1.6 mW/cm^2^) Blue (455–460 nm, 3.84 mW/cm^2^) Green (515–530 nm, 4.5 mW/cm^2^) Red (655–660 nm, 4.07 mW/cm^2^) Near-IV (750 nm, 3.1 mW/cm^2^) 30 min dark (ads) + 1–2 h light (deg) Single-solute	BiOCOOH adsorbs at ≈367 nm (UV) but the incorporation of 1–5% CQDs redshifts the adsorption spectra and allows superior visible light adsorption (E_g_ = 3.42–2.81 eV) Under visible light BiOCOOH/CQD with 2.0 wt.% of CQD reaches 98% DCF degradation while the bare BiOCOOH only attains 51.5% degradation, the commercial P25 degrades less than 15% of the DCF CQD on the surface of BiOCOOH operates as photosensitizers O_2_^•−^ and holes contribute for the higher BiOCOOH/CQD photoactivity In 2 h under visible light BiOCOOH/CQD mineralize, detoxify (*V. fisheri*, *D. magna,* and *D. subspicatus* tests) and dechlorinate DCF 90% DCF degradation after 4 CQD/BiOCOOH reuses under visible light Intermediaries identified and degradation mechanisms proposed	[138]
DCF	TiO_2_{001}/N-CQD TiO_2_{101}/N-CQD TiO_2_(P25)/N-CQD	Composites: 0.25 g TiO_2_ with 0.5–8.0 cm^3^ of N-CQD 10 g/dm^3^ [Catalyst] = 1 g/dm^3^ [DCF]_0_ = 10 mg/dm^3^ pH 3–11 Visible > 420 nm (58.6 mW/cm^2^) Broad spectrum: simulated sunlight, > 290 nm (60.0 mW/cm^2^) near IR (750 nm LED, 36.9 mW/cm^2^) UV (365 nm LED, 35.2 mW/cm^2^) Photocatalysts (LED): 450 nm (71.6 mW/cm^2^), 520 nm (28.0 mW/cm^2^) and 660 nm (67.4 mW/cm^2^) 30 min dark (ads) + 1 h light (deg) Single-solute Deionized water, river water, sea water, wastewater effluent	Under visible light TiO_2_{101}/N-CQD and TiO_2_{001}/N-CQD degrade ≈80% and >90% DCF in 1 h while TiO_2_(P25)/N-CQD and bare TiO_2_ degraded less than 20% of DCF (0–7.5% of adsorption contribution) TiO_2_{001}/N-CQD higher performance under broad-spectrum irradiation attributed to high oxidation activity of exposed {001} facets DCF photodegradation kinetic rate of depends on the CQDs amount Additional OH^●^ and O_2_^●−^ after coupling TiO_2_{001} with N-CQD (compared with bare TiO_2_{001}) TiO_2_{001}/N-CQD has lower estimated E_g_ (2.7 eV) than TiO_2_{001} (3.1 eV) being beneficial for sunlight harvest, a direct Z-scheme heterojunction is the most probable charge transfer mechanism 8 intermediates identified and the degradation mechanism of DCF with TiO_2_{001}/N-CQD under visible light is proposed After 1 h visible light irradiation with TiO_2_{001}/N-CQD 90% of DCF was removed but only 50% TOC removal was attained pH increase slows DCF photocatalytic degradation Transition metals and humic acids in water restrain DCF degradation, but assays in natural waters show that DCF photodegradation is only slightly inhibited compared to deionized water After 4 reuse cycles under visible light TiO_2_{001}/N-CQD still degrades 80% of DCF	[139]
DCF	g-C_3_N_4_/CQD g-C_3_N_4_	Composites and g-C_3_N_4_ prepared via polymerization (0.02, 0.05, 0.1 and 0.2 cm^3^ of CQD) [Catalyst] = 0.2 g/dm^3^ [DCF]_0_ = 10 mg/dm^3^ pH 5–9 Visible light (300 W Xe lamp, 400–700 nm, 150 mW/cm^2^) 30 min dark (ads) + 1 h light (deg) Single-solute	g-C_3_N_4_/CQD with lower amounts of CQDs (0.02 and 0.05 cm^3^) have enhanced visible light absorption compared to g-C_3_N_4_ (adsorption edges are 485 nm and 550 nm and E_g_ 2.55 eV and 2.25 eV), CQD suppress g-C_3_N_4_ emission peak suggesting improved e^−^/h^+^ separation Higher CQDs amounts enlarge the absorption towards IR, by acting as active sites they have a shield effect and inhibit photocatalysis Due to high e^−^/h^+^ recombination rate bare g-C_3_N_4_ only removes 19.3% of DCF after 1 h irradiation, composites attain removal efficiencies 49.5–100% with g-C_3_N_4_/CQD-0.05 presenting the highest and fastest (7.4 × 10^−3^ min^−1^ vs. 4.9 × 10^−3^ min^−1^ for g-C_3_N_4_) removal of DCF Faster removal at alkaline pH (0.47 min^−1^ pH 9 vs. 3.2 × 10^−2^ min^−1^ pH 5) OH^●^ and O_2_^●−^ contribute to DCF degradation: O_2_^●−^ plays the dominant role thus the capture of e^−^ by O_2_ seems to be the rate-determining step DCF degradation not dependent on h^+^ and DCF absorption spectrum mainly in the UV region thus a photosensitation-like degradation mechanism is proposed 8 intermediates detected and three main degradation routes proposed TOC removal up to 54% for 1.5 h of irradiation thus DCF might be mineralized for longer irradiation times: in general intermediates are less toxic than DCF but they are still classified as very toxic After 5 reuse cycles g-C_3_N_4_/CQD attained 90% removal for DCF	[140]

ads–adsorption, deg-degradation

**Table 8:** Photocatalytic degradation of CBZ and SMX by semiconductor/char.

PhC	Catalyst	Experimental Conditions	Concluding Remarks	Ref.
SMX	Zn-TiO_2_/biochar TiO_2_ TiO_2_/biochar	Sol-gel method: Zn(NO_3_)_2_ (1%, 10% & 15%), 1 g of pretreated reed straw biochar and 20 cm^3^ tetrabutyl titanate (calcined at 300 °C) [Catalyst] = 0.625–2.5 g/dm^3^ [SMX]_0_ = 10 mg/dm^3^ pH 2.01–10.97 Simulated visible light (50 W,Xe lamp, >420 nm) 30 min dark (ads) + 3 h light (deg) Single-solute Deionized water & spiked river water	SMX removal: Zn-TiO_2_/biochar > TiO_2_/biochar > TiO_2_, up to 80% SMX removal in 3 h and ≈55% COD Zn-TiO_2_/biochar with Ti/Zn mass ration of 10:1 is the best performing material for degrading SMX (0.085 min^−1^) pH 5 and 1.25 g/dm^3^ catalyst allow faster and higher SMX removal Assays in spiked river water reveal decreased efficiency of Zn-TiO_2_/biochar (53.83%) compared with deionized water (>80%) Common anions (e.g., SO_4_^2−^, Cl^−^ and NO_3_^−^) have an inhibition effect on SMX degradation possibly due to OH^●^ trapping or capture of h^+^ (hinder OH^●^ production) Zn-TiO_2_/biochar photoactivity slightly decreases after 1^st^ cycle but remains almost constant in the 4 next ones SMX photodegradation mechanism by Zn-TiO_2_/biochar under visible light proposed, degradation products identified and 4 possible degradation pathways proposed	[145]
CBZ	Fe_3_O_4_/BiOBr/Biochar Fe_3_O_4_/BiOBr BiOBr	One-step hydrolysis method: 5%, 10%, 20% and 30% (in wt.%) reed straw biochar [Catalyst] = 1 g/dm^3^ [CBZ]_0_ = 10 mg/dm^3^ pH 3–10 Visible LED light (50 W, 475 nm) 1 h dark (ads) + 3 h light (deg) Single-solute	Amounts of Fe_3_O_4_ and biochar influence the photoactivity of the composite for CBZ degradation under visible light during 3 h: 95% removal attained for 10% biochar and 0.05 g of Fe_3_O_4_ At pH 6 Fe_3_O_4_/BiOBr/biochar is the best performing photocatalyst: highest rate constant (0.01777 min^−1^), CBZ removal similar to that of BiOBr (95%) associated to mineralization degree of 70% (only ≈30% for BiOBr and ≈55% for Fe_3_O_4_/BiOBr) Optimum catalyst dosage is 1.0 g/cm^3^ and solution pH is 7.1: faster (0.02292 min^−1^) and almost 100% CBZ removal Cl^−^ and SO_4_^2−^ inhibit CBZ photodegradation Humic acids role depends on concentration: low conc. – detrimental, high conc. – photosensitizers promoting CBZ photodegradation After 5 reuse cycles Fe_3_O_4_/BiOBr/biochar degrades 90% CBZ under visible light, after 4 reuse cycles mineralization > 60% OH^●^, h^+^ and O_2_^●−^ seem to take part in the CBZ photodegradation with a suggestion that oxygen radicals play the most important role 15 main intermediates and 2 ring-rupturing products identified, 4 possible reaction pathways proposed	[146]
CBZ	TiO_2_/biochar TiO_2_ biochar	Sol-gel method: TiO_2_/coconut shell powder char (34.04 cm^3^ titanium-n-butoxide and 60 – 120 g biomass) Pellets: mixing composites with 10% wt. of wheat flour, calcined at 500–800 °C for 2 h [Catalyst] = 60–120 g/dm^3^ [CBZ]_0_ = 10 mg/dm^3^ 24 g/dm^3^ O_2_ flow (bottom-to-top) pH 3–11 UV-C (10.5 mW/cm^2^) 60 min light (ads+deg) Single-solute & deionized water	Composites have higher adsorption and photoactivity than TiO_2_ or char Composite with 100 g biomass calcined at 700 °C has attains 98% CBZ removal possibly due to higher surface area and lower crystallite size	[147]
CBZ	TiO_2_/biochar TiO_2_ GAC biochar	Similar to ref. [147] [Catalyst] = 60–140 g/dm^3^ [CBZ]_0_ = 10 - 50 mg/dm^3^ 6–24 g/dm^3^ O_2_ flow pH 3–11 UV-A (4.2 mW/cm^2^) and UV-C (10.5 mW/cm^2^) 60 min light (ads+deg) Single-solute & deionized water	Almost constant (0.05 min^−1^) CBZ degradation rate for pH 3 to 11, attaining 89.2–94.4% CBZ removal O_2_ flow increase improves the CBZ photodegradation rate constant more than 1.6 times (0.025 to 0.042 min^−1^) After 1 h of UV-C irradiation GAC and composite remove, respectively, ≈90% and ≈99% CBZ while TiO_2_ and coconut shell powder only remove 35–42% of CBZ Enhanced adsorption+degradation with the composite attributed to the surface area that allows adsorbing CBZ that is further photodegraded by the reactive oxygen species produced by the semiconductor Composite has superior recycling performance over 11 reuse cycles	[148]

ads–adsorption, deg-degradation
